# Chemically Specific Multiscale Modeling of Clay–Polymer Nanocomposites Reveals Intercalation Dynamics, Tactoid Self-Assembly and Emergent Materials Properties

**DOI:** 10.1002/adma.201403361

**Published:** 2014-12-09

**Authors:** James L Suter, Derek Groen, Peter V Coveney*

**Affiliations:** Centre for Computational Science, University College London20 Gordon Street, London, WC1H 0AJ, UK

**Keywords:** multiscale modeling, clay–polymer nanocomposites, intercalation dynamics, tactic self-assembly, materials properties

## Abstract

A quantitative description is presented of the dynamical process of polymer intercalation into clay tactoids and the ensuing aggregation of polymer-entangled tactoids into larger structures, obtaining various characteristics of these nanocomposites, including clay-layer spacings, out-of-plane clay-sheet bending energies, X-ray diffractograms, and materials properties. This model of clay–polymer interactions is based on a three-level approach, which uses quantum mechanical and atomistic descriptions to derive a coarse-grained yet chemically specific representation that can resolve processes on hitherto inaccessible length and time scales. The approach is applied to study collections of clay mineral tactoids interacting with two synthetic polymers, poly(ethylene glycol) and poly(vinyl alcohol). The controlled behavior of layered materials in a polymer matrix is centrally important for many engineering and manufacturing applications. This approach opens up a route to computing the properties of complex soft materials based on knowledge of their chemical composition, molecular structure, and processing conditions.

## 1. Introduction

The search for new materials with properties optimized for particular applications is a worldwide endeavor. In many domains, discoveries are made serendipitously in the laboratory; it may take decades before such materials can be developed and manufactured to meet real-world requirements. Materials optimization would be considerably assisted by reliable modeling tools that relate the chemical composition of a material to its properties, which are often determined on far greater length and time scales than those pertaining to the atoms and molecules of which it is comprised. Thus, the theoretical challenge is the very substantial one of linking the microscopic level to higher levels of description, transferring the physicochemical representation from the lowest level of concern to the highest, so that predictions of the materials properties faithfully reflect the underlying chemical characteristics. The purpose of this paper is to demonstrate such a multiscale approach to calculating materials properties of soft-matter systems, in particular clay–polymer nanocomposites. Their durability and strength, coupled to low density hold out the potential, so far only partially realized, for wide-ranging applications in engineering and manufacturing.[1,2] We first proceed to concisely introduce existing research on multiscale modeling and clay–polymer nanocomposites, and then present our new multiscale approach.

## 2. Clay–Polymer Nanocomposites and Multiscale Modeling

**Figure 1:**
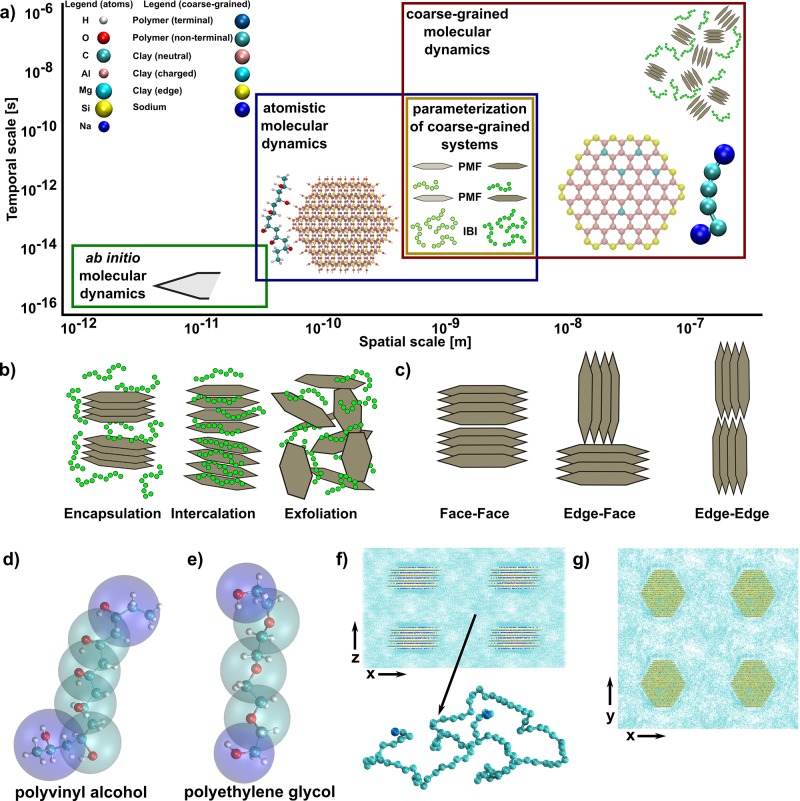
a) Schematic representation of our multiscale scheme. We use ab initio quantum mechanical calculations to calculate potentials of sheet edges, and all-atom classical molecular-dynamics simulations to model the local behavior of the system. We determine the parameters and potentials for the coarse-grained system using iterative algorithms, matching to the behavior of the all-atom system. The methods include matching to structural properties such as radial distribution functions or density profiles perpendicular to the clay sheet (iterative Boltzmann inversion (IBI)) and through potential of mean force (PMF) calculations when an unbiased classical molecular-dynamics simulation does not sample enough configuration space to define a potential using IBI (see Supporting Information for more details). The largest scale is then resolved using coarse-grained molecular dynamics. b) Three key configurations observed in clay–polymer interactions: encapsulated (left), intercalated (middle), and exfoliated state (right), where the gray hexagonal platelets are representative of the clay sheets. c) Schematic overview of several possible tactoid alignments, including face–face (left), edge–face (middle) and edge–edge (right). The final microstate depends not only on the interactions present, but also on the processing conditions. d) Mapping of coarse-grained particles to atoms for PVA. The coarse-grained particles are indicated by the larger semi-transparent spheres, blue for terminal monomers and green for non-terminating monomers. e) Same as (d), but for PEG. f) Starting arrangement of the 8 tactoids in our coarse-grained simulations shown in the *xz* plane. g) Same as (f), but shown in the *xy* plane.

Clays are naturally found as tactoids, which may contain from a few to as many as one thousand stacked sheets. The properties of a clay–polymer nanocomposite are thought to be associated in some way with the extent to which these clay tactoids remain intact, or exfoliate and then disperse in the polymer matrix during processing[3–5] (see **Figure** **1**b for an overview of three common configurations and Figure 1c for three common clay-sheet self-assembly patterns).

Clays, such as montmorillonite (MMT), consist of nano­meter-scale sheets of aluminosilicates. The sheets possess dimensions of 1 nm thickness and are 100 to 500 nm in diameter, resulting in platelets of very high aspect ratio. On the level of an individual platelet, the clay minerals are very stiff, with estimates of 160–400 GPa:[6,7] transfer of stress from the polymer to the mineral – in part due to the large interfacial area between the mineral and polymer – results in increased mechanical strength, whereas the flexibility of the polymer prevents the composite from being brittle. Clay–polymer nanocomposites with less than 5 wt% clay, in which, ideally, the clay sheets are completely exfoliated and homogeneously distributed with random orientation throughout the polymer matrix, were discovered first by Okada and co-workers at Toyota Central Research in 1985[8] and offer higher stiffness, fire retardency, and better barrier properties than the parent polymer.[1,9,10]

Thus, one aim is to control the dispersion of the clay sheets on the microscale for targeted properties. The thermodynamic propensity for clay sheets to exfoliate depends on the free energy required to separate the clay sheets, the free energy to create voids in the polymer matrix, and the free energy of interaction between the clay sheets and the polymer.[5] One approach is to lower the free energy required to cleave the clay sheets. In natural clays, substitutions in the clay framework (Si for Al and Al for Mg) are commonly found; as a result, charge-balancing counterions, such as Na^+^ and Ca^2+^, reside between the clay sheets. The variations in chemical composition can be used to select a clay mineral with favorable cleavage energy; alternatively, the natural counterions can be replaced with low-molecular-weight quaternary ammonium compounds that increase the basal-plane spacing. These surfactants can also facilitate intercalation by increasing the interaction between the polymer and the clay surface. This is especially important for hydrophobic polymers such as polyethene, polyproylene, polystyrene and poly(methyl methacrylate) (PPMA), which would otherwise be immiscible with clay particles.[11] There is ongoing effort to tune the dispersion of clay platelets through the composition of the surfactants;[11] Heinz et al. have quantified these aspects for nanocomposites of octadecylammonium and montmorillonite[12] through the calculation of cleavage energies by molecular simulation.

At high clay volume fractions, one seeks ordered structures, mimicking the microstructures of nacre[13] or mother of pearl,[14] in the attempt to produce durable and rigid composites. At low clay volume fractions, one aims for complete dispersion to increase the interfacial area and produce lightweight and very strong materials. Notable examples of low-volume fraction clay–polymer-nanocomposite applications occur in automotive applications,[8,15,16] packaging,[1] biotechnology,[5,17] regenerative medicine,[18] and medical devices.[1] Alternatively, in oil-field applications the swelling of clay minerals through the adsorption of water can result in well-bore instability; organic polymers and surfactants incorporating appropriate functional groups are used to reduce swelling.[19]

The potential large-scale engineering and manufacturing of clay–polymer nanocomposites remains challenging due to the paucity of laboratory-based searches in finding appropriate combinations of clays and polymers, processing conditions, and optimal clay volume fractions (Figure 1);[8] all are factors deemed essential for obtaining desirable materials and structural properties. In addition, we do not yet fully understand how the interfacial bonding and structure at the nanoscale in these nanocomposites lead to observed properties. Multi­scale modeling has received significant attention over the past 15 years[20–24] and systematic, theory-driven multiscale approaches would allow us to explore these questions by computational means, accelerating the route from concept to real applications. In Figure 1a, we illustrate the multiscale simulation scheme that we demonstrate in this paper: for small length and timescales (<10^−9^ s and 10^−8^ m) we can use techniques to solve an electronic description of the system, at intermediate length and timescales (<10^−7^ s and 10^−6^ m) we can use molecular dynamics (MD), where atoms are represented by classical particles described by interaction-energy terms (a forcefield), while for longer time and length scales (<10^−5^ s and 10^−5^ m) we can use coarse-grained MD, where several atoms are grouped into single particles.

The coarse-graining of polymer systems from all atom models has advanced, primarily for synthetic polymers in homogeneous environments,[25] and for the simulation of lipids,[26,27] proteins,[28–30] and nucleic acids.[31,32] In addition, coarse-grained MD has been applied to model polymer interactions close to nonclay solid surfaces. Johnston and Harmandaris[3] provide a comprehensive review of this field, from which we highlight a few key recent contributions. De Virgilis et al. applied coarse-graining to model a polymer melt near an adhesive solid substrate,[33] while Wallace and Sansom invoked it to model detergents interacting with carbon nanotubes.[34] In addition, Johnston and Harmandaris investigated the behavior of polymer–gold systems using a hierarchical multiscale approach, which includes coarse-grained simulations of individual (rigid) sheet interfaces.[35] Several important methodologies have emerged to obtain accurate potentials for coarse-grained particles, which allow these coarse-grained models to address many properties of atomistic models with high accuracy. These include the application of iterative Boltzmann inversions (IBI) to match coarse-grained potentials to atomistic radial distribution functions (RDFs),[36] and coarse-graining techniques that rely on calculating the potential of mean force between particle types.[37] A major emerging area in materials modeling is polymer–graphene nanocomposites, which can be created using melt intercalation (see for example refs. [38,39]]. and for which Potts et al. provide a comprehensive review of the experimental activities.[40] Rissanou and Harmandaris have conducted a preliminary modeling study of polymer–graphene nanocomposites[41] using all-atom simulations, and Merlet et al.[42] have applied coarse-grained MD to model ionic liquids interacting with graphite surfaces. However, to our knowledge, no modeling approach has been able to capture the dynamical intercalation mechanisms and the ensuing large-scale self-assembly processes that dominate the behavior of these systems. Indeed, multiscale approaches of the kind presented here are essential to tackle this major scientific challenge.[43]

### 2.1. Experimental Studies of Clay–Polymer Nanocomposites

There is extensive experimental literature on polymer nanocomposites available, from which we highlight a few recent contributions of particular relevance to our work. Paul and Robeson present a review of nanocomposites and their properties.[15] They compare the properties of nanoscale dimensions relative to larger scale ones, aiming to understand the property change as particle dimensions decrease to the nanoscale level. At these sizes, the increase in surface area is up to three orders of magnitude larger than conventional composites, and the clay particles are of a similar size to the radius of gyration *R*_g_ of the polymer (typically 3–30 nm). The addition of even a small amount of clay particles renders a very large fraction of the polymer in an “interface” region, where the properties of the polymers are perturbed compared with those in the bulk (i.e., away from the surface) due to interactions with the clay surface. The percolation threshold can be reached at <1–5 vol% clay.[44] The polymer–clay interactions are therefore highly important, as so much of the polymer is in the “interface” region. Paul and Robeson[15] report that the increased mechanical properties of the composite can be explained from the reinforcement of the clay particles by using composite theories, taking into account the high aspect ratio and nanoscale dimensions. This indicates that, in nanocomposites, the transfer of stress onto the stiffer clay particles is highly efficient. There is no evidence that changes in the polymer due to the “interface” layer, such as the temperature of crystallization *T*_c_ or the glass-transition temperature *T*_g_, influence the materials properties.

Bousmina[45] discusses the physics of intercalation and exfoliation processes in polymer nanocomposites. They found that, on the basis of mean-field equations, clay particles would not intercalate if they were untreated by surfactants unless there are highly favorable interactions with the polymer. From experiment, they concluded that exfoliation of surfactant-treated clay, with its expanded clay-layer separation, requires a balance between the mechanical stress required to “peel” off the clay layers and the relatively low levels of shearing required to allow polymer molecules to diffuse between the clay sheets. Once the polymers had diffused into the clay interlayer, high shear rates could then further exfoliate the clay sheets. This indicates that, while intercalation/exfoliation can be thermodynamically spontaneous with highly interacting polymers, mechanical stresses are still required to exfoliate even surfactant-treated clays with hydrophobic polymers.

Several groups have reviewed the possible applications for clay–polymer nanocomposites. These include a recent review by Ray,[1] which systematically covers the application areas for clay–polymer nanocomposites, and identifies a recent trend in research towards multifunctional and biodegradable nanocomposites. Heinz provides a comprehensive review of clay–polymer nanocomposites in the context of biotechnology.[5] For further details on the experimental studies of clay–polymer nanocomposites, a significant number of excellent reviews are available.[9,10,46,47]

### 2.2. Modeling Clay–Polymer Nanocomposites

Hitherto, modeling work within the clay–polymer-nanocomposites domain has primarily been performed on isolated length and time scales.[48] A large amount of research has been performed at the classical atomistic molecular-dynamics level, where accessible length and timescales (see Figure 1a) allow the study of the structural and dynamical behavior within the clay interlayers. In such an approach, periodic boundaries are placed on the clay sheets, thereby effectively simulating an infinite clay sheet. Several reviews exist in the literature on the use of classical atomistic MD for clay systems;[48–52] here, we highlight the parameters required for simulation and studies that are of importance to the research presented.

There are several forcefields for classical molecular-dynamics simulations in common use for clays that are parameterized, fully or in part, from electronic (quantum) descriptions. These include the ClayFF forcefield by Cygan et al.*,*[53] the forcefield of Teppen et al.[54] and the recently developed INTERFACE forcefield by Heinz et al.[55] Each forcefield has been fitted to selected quantities, which may be from electronic-structure calculations (such as the electrostatic potential near the clay surface) or experimental quantities (for example, the INTERFACE forcefield is fitted to electron densities, cleavage energies, and surface tensions). Cygan et al. have produced a comprehensive review of classical molecular models and forcefields for clays (and other layered materials).[49] Examples of simulations at the classical molecular-dynamics level include models that quantify the bending of clay sheets,[56] capture the dynamics of surfactants on clay mineral surfaces,[49] and a molecular-mechanics/molecular-dynamics model that predicts the binding energy of these nanocomposites.[57]

A few groups have adopted multimodel approaches to simulate clay–polymer nanocomposites. These include Scocchi et al.*,*[58] who combined an all-atom molecular-dynamics simulation with a dissipative particle-dynamics simulation to investigate the material properties of Nylon-6 between two rigid sheets of montmorillonite clay, and Sikdar et al.,[59] who combined MD with a finite element study, which thus incorporated an additional polymer phase representing the interfacial region. Sinsawat et al.[60] studied the behavior of clay sheets using coarse-grained MD, observing changes in self-assembly behavior when the generic interaction potentials were modified. Some other approaches have been applied to model intercalation, for example Vaia et al. applied lattice models to simulate melt intercalation in surfactant-treated clay–polymer nanocomposites.[61,62]

We highlight publications that calculate the materials properties of clay–polymer nanocomposites. At the atomistic level, the elastic constants for a single sheet of montmorillonite were calculated by simulating the response of the clay to deformation;[7] a value of approximately 400 GPa was determined for the in-plane Young's modulus. In our previous work, we determined the elastic constants of a montmorillonite sheet through thermal fluctuations of the clay sheet,[63] through which we determined a Young's modulus of 260 GPa. In more-recent work, Fu et al. simulated the bending of a clay sheet until fracture occurred. The authors found that clay sheets could bend significantly before failure (a curvature corresponding to a bending radius of 3 nm).[56] This indicates that clay sheets can possess high bending energy, a feature seen in transition electron microscopy (TEM) of montmorillonite embedded in amine-cured epoxy and in silk elastin polymer matrices.[11] In our own work, we observed that montmorillonite clay could withstand high strain levels before buckling occurs.[64] For clay–polymer nanocomposites, in our previous work, we calculated the elastic properties of a montmorillonite–poly(ethylene glycol) (PEG) composite, from which we could partition the response from the clay and the adsorbed polymer layers.[65] We observed that the layer of PEG molecules adsorbed on the surface had different conformations to polymer molecules further from the clay surface; these molecules had a small but significantly higher modulus than polymer molecules far away from the clay surface.

All the previous simulations utilized periodic boundaries on the clay sheet. This prevents the study of the edges of the clay sheets and the microstructure of clay platelets. To overcome this, Sheng et al. used representative volume elements (RVE) to simulate an array of dispersed platelets in a polymer matrix, using elastic constants for the clay (unintercalated and intercalated) from molecular-dynamics simulations, while treating the clay stack as an “effective-particle”.[66] They found that clay particles significantly enhance the modulus of the composite at low volume fractions, although the expected large enhancement between intercalated and exfoliated clay sheets was not seen. This reflects the fact that, even when intercalated, most of the polymer can still be considered to be within the “interface” region. Their model assumes uniform, well-aligned and perfectly bonded clay particles.

What is required is an intermediate level, where the microstructure, on the level of multiple tactoids, is determined by the underlying chemical interactions, and the calculation of materials properties reflects the interfacial bonding between the clay and polymer and the nanoscale geometry of the clay interlayer spacing. Here, we describe a multiscale modeling scheme, which allows us to make such macroscopic predictions for clay–polymer materials by computational means, while including the molecular and nanoscale processes that underpin them (Figure 1). We extract essential information pertaining to the parameter-free quantum-mechanical description and transfer it, via all-atom classical MD, to a coarse-grained dynamical description in which the modeled material retains the essential features associated with its chemical specificity. From there, we study the dynamics of the intercalation process, including the mechanism of entry in intercalation, as well as the process of tactoid self-assembly. Based on these simulations, we then investigate the resultant unusual materials properties.

### 3. Description of the Clay–Polymer System

In this study, we consider the archetypical swelling clay, montmorillonite (MMT), which possesses a negative charge due to isomorphic substitutions in the clay framework. We consider only charge sites in the octahedral layer of the clay, where Al^3+^ ions are replaced by Mg^2+^ ions, which are the majority of charge sites for such clays. One in every 9 Al ions is replaced by an Mg ion. These charge sites are counterbalanced by sodium ions, resident in the intersheet spacing. The charge sites are distributed at both the classical and coarse-grained levels, according to a homogeneous distribution of charge, as inferred from ^29^Si NMR studies.[67] For mapping the classical all-atom system to the coarse-grained (CG) equivalent, each CG clay particle position is coincident with either an Al or Mg ion position in the octahedral layer of the clay. Similarly, Na CG particles correspond to the positions of the Na^+^ ion. In total there are 4 CG clay types: neutral clay (corresponding to a Al^3+^ octahedral ion), charged clay (Mg^2+^), edge clay (five coordinate Al^3+^), and sodium (Na^+^). Bonds, as well as terms reflecting in-plane and out-of-plane angles were matched to atomistic simulations (see the Supporting Information for more details). In our models, we wish to reproduce the conditions of melt intercalation. This process involves increasing the temperature above the softening point of the polymer, so that the polymer and clay are mixed together, whereupon polymer molecules may penetrate into the clay interlayer gallery. We therefore set the temperature in our simulations to 500 K. We have considered two hydrophilic polymers, poly(ethylene glycol) (PEG) and poly(vinyl alcohol) (PVA), which do not require modification of the clay surface to form composites.[1] We created two models for each polymer: a length of 100 monomers (“long” type) and a polydisperse mixture of 31 to 35 monomers (“short” type). The latter was chosen such that the number of CG particles was the same for both models, with three times the number of molecules in the “short” case. Each monomer unit corresponds to a polymer CG particle. Figure 1e and 1f depict the mapping of an all-atom polymer onto a CG particle, while the derivation of the CG inter- and intramolecular interaction terms is given in the Supporting Information. These four configurations allow us to explore systems with different lengths and types of polymers, and allow us to compare our models with experimental observations for both PVA–clay and PEG–clay nanocomposites.

Our systems were created by constructing a clay tactoid comprising four hexagonal sheets, each composed of (110), (010), and (001) surfaces, with lateral dimensions measuring approximately 98 Å by 104 Å, each sheet separated by 9.5 Å. The tactoid was inserted into a simulation box, lying in the *xy* plane, with the periodic boundaries selected to be no less than 90 Å to the closest atom; the resulting simulation box dimensions were 201 Å × 201 Å × 120 Å. Polymer molecules were added to this system using a Monte Carlo growth method to a required density that matched that of the bulk atomistic system, thereby creating a coarse-grained polymer in the required molten state. This system was further replicated in the *x*, *y*, and *z* directions by 2 × 2 × 2 respectively to form a dispersed 8-tactoid system, with dimensions 402 Å × 402 Å × 240 Å (see Figure 1g for a graphical overview). The clay–PVA systems contain 388 704 coarse-grained particles in total, corresponding to 2 813 664 atoms. Both PEG systems contain 402 304 coarse-grained particles, corresponding to 2 857 248 atoms.

**Figure 2:**
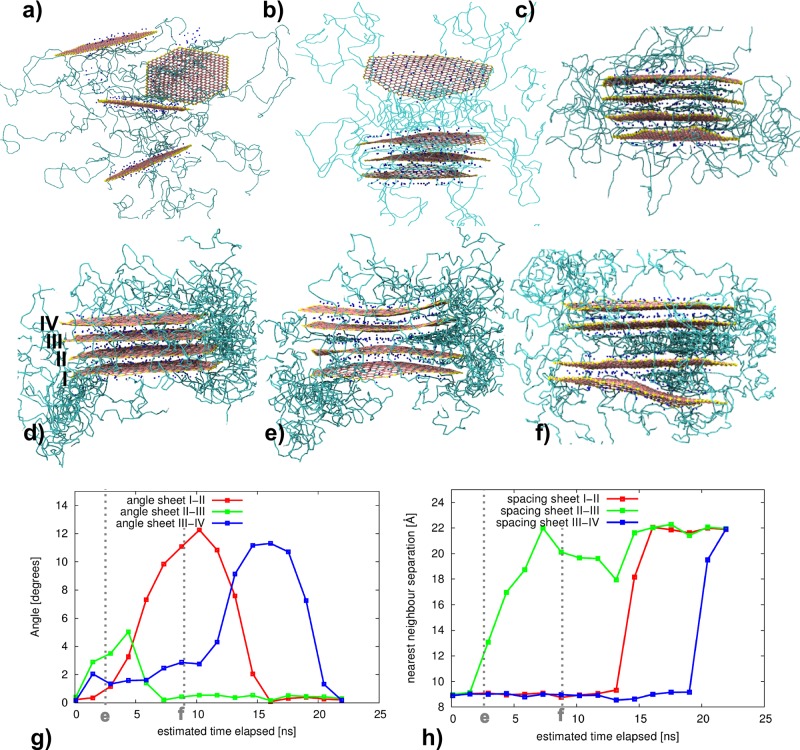
Overview of two intercalation processes for selected tactoids, one in the “long” PEG system (a–c) and one in the “long” PVA system (d–f). The polymer molecules are represented by the green bonds; for the clay CG particles the colors are: neutral clay is pink, charged clay is cyan, edge clay is yellow, and sodium is blue. Only selected polymer molecules are shown to aid visualization. In the “long” PEG system, which starts as a single tactoid in an exfoliated state (a), the clay sheets gradually self-assemble into an intercalated clay tactoid (see (b) for an intermediate snapshot and (c) for the final state). A monolayer of PEG remains between the MMT sheets, resulting in a clay tactoid with an average *d*-spacing of about 16 Å. In the “long” PVA simulation the clay sheets, initially unmixed (d) begin to undulate, which initially leads to an opening in the center of the tactoid and the entrance of PVA molecules between the central sheets (e). As time progresses, the outer sheets further undulate and, through the formation of a solitary wave that propagates along the clay platelet, intercalation occurs in the remainder of the tactoid as well (f). g) We present the nearest-neighbor distance between each adjacent pair of sheets in the tactoid shown in Figure 2d–f as a function of the simulated time in nanoseconds. h) We present the angle between the planes that are fitted to each of the clay sheets. This angle allows us to determine whether adjacent clay sheets are fully parallel (angle = 0°) or whether the tactoid has opened up, with a pair of clay sheets having a greater separation distance on one side than on the other. We observe that increases in *d*-spacing are preceded by changes in the angle between adjacent clay sheets, and conclude that sheet bending triggers these intercalation events.

In addition to the aforementioned systems, which start with unintercalated tactoids, we have also modeled two systems (“long” PEG and “long” PVA), starting from an exfoliated state, where 4 clay sheets were randomly distributed in a simulation box of 201 Å × 201 Å × 120 Å (ensuring no clay sheets are overlapping). An illustration of the exfoliated starting structure is shown in **Figure** **2**a.

We ran each CG simulation for more than 1 μs at a temperature of 500 K to simulate melt intercalation. This allows us to extract the behavior on both the intratactoid level, including the dynamics of intercalation, and subsequently on the intertactoid level (i.e., self-assembly of the tactoids, shown in Figure 1).

## 4. Multiscale Modeling Approach

The purpose of this section is to describe the construction of the coarse-grained potentials used in this study, and how they are applied to investigate these materials. Our approach is schematically described in Figure 1a and amplified within this section. The potentials were derived from simulations performed at three separate levels, ab initio, all-atom MD and coarse-grained MD, which we now discuss.

### 4.1. Ab Initio

Firstly, we derived classical atomistic clay potential parameters from the electronic structure, calculated using quantum-mechanical density functional theory (DFT). A model of the montmorillonite clay (110) edge was created through cleaving the neutral crystal structure in the required Miller plane; dangling bonds were saturated using hydrogen atoms or hydroxyl groups. This system contained 184 atoms and was simulated in vacuum, using a simulation box of dimensions 22 Å × 10.24 Å × 17.5 Å. The formula for the (110) clay-edge simulation is Al_16_Si_32_(OH_8_)O_80_H_8_(OH)_16_. Plane-wave density functional theory calculations were performed to determine the electronic structure, which was used to calculate the electrostatic potential. The terminal edge atoms are not currently described by the ClayFF forcefield, a widely adopted forcefield pertaining to clay minerals and their interfaces, which we use in this work.[53] The atomic charges on the terminating hydrogen and hydroxyl groups were assigned using the restricted electrostatic potential technique. These charges were then further refined to ensure that the radial distribution functions of the terminal atoms and surrounding water, simulated using classical MD with these parameters, were in good agreement with those simulated using ab initio MD. The final charges were: terminal oxygen −0.925, terminal hydrogen 0.4.

### 4.2. Deriving Coarse-Grained Parameters

The parameterization, verification, and validation of the coarse-grained potentials from more-accurate methods are described in the Supporting Information. Here, we summarize the main aspects. To derive the CG molecular-dynamics parameters, we require a variety of techniques. Our approach was to parameterize in a step-wise manner: CG forcefield parameters were determined through successive simulations at the atomistic molecular-dynamics level, the simulations chosen such that no more than three CG interaction parameters were derived in any one fitting procedure. In this way, we avoid the artifacts caused by deriving CG potentials from incomplete sampling at the classical MD level, which can often occur when many parameters are fitted to a single atomistic simulation. For example, polymer–polymer CG parameters were first derived from an atomistic simulation of pure polymer; this was followed by polymer–clay interactions, where only the polymer–clay CG interactions were obtained while the polymer–polymer interactions, derived previously, were kept constant (see the Supporting Information for a workflow diagram of this procedure). We used the LAMMPS MD code to perform all our classical atomistic and coarse-grained simulations.[68] Each such simulation at the atomistic level was performed in the *NPT* ensemble at 500 K and at 1 atmosphere, using the particle–particle/particle mesh (PPPM) algorithm[69] for the electrostatics with a 10 Å cutoff both for the real-space part of the Ewald sum and for the Lennard–Jones potentials. All the atomistic simulations used the ClayFF[53] and CVFF[70] forcefields to describe the clay and the polymers respectively. Previous simulations using this combination of forcefields have shown good agreement with basal-plane spacings, interlayer density, and chain conformations.[71–73] A combined Nosé–Hoover thermostat and barostat was used to keep the temperature and pressure constant.

The non-bonded parameters between polymers were derived using iterative Boltzmann inversion (IBI),[74] which ensures the radial distribution functions are matched at the atomistic and CG levels. Within each IBI iteration, we used the pressure correction of Wang et al.[36] to ensure the pressure of the CG simulation was within one standard deviation of the corresponding atomistic simulation.

The IBI method is difficult to converge when used to create interaction potentials in the vicinity of surfaces. In this case, the isotropic environment normally encountered in the IBI method is not found; instead, a molecule adsorbing on the surface interacts with a large number of surface atoms at varying distances. Indeed, the presence of interfaces in the polymer–solid system complicates the derivation of systematic CG models in this way.[3] Matching to the atomistic density profile perpendicular to the clay sheet is a more-natural choice for interfacial phenomena, as the density of adsorbed and successive layers of the polymer will be consistent. We therefore extended the IBI method to match to this function rather than to radial distribution functions. As shown in the Supporting Information, the CG interaction potentials derived to match the density profiles were also in good agreement with atomistic radial distribution functions. To derive interaction potentials between CG particles that have high levels of attraction, we require an alternative approach to IBI, as a single atomistic simulation will only explore the highly favorable regions of phase space, which are not enough to derive a complete CG interaction potential. These classes of CG potentials include clay–clay, sodium–clay and sodium-charged clay particle interactions. To overcome this limitation, we matched the potential of mean force (PMF) between the atomistic and coarse-grained levels, calculated using umbrella sampling techniques, and recombined using the weighted histogram analysis method (WHAM).[75] In the Supporting Information, we show the agreement obtained between CG and atomistic radial distribution functions, density profiles and PMFs for each derived CG interaction. It is also shown that there is agreement between the atomistic and coarse-grained simulations of the interlayer spacing of montmorillonite as a function of increasing polymer content, a powerful validation of the coarse-grained parameterization.

### 4.3. The Coarse-Grained System

Our coarse-grained simulations use non-rigid clay sheets that have elastic properties, consistent with our earlier work[19,63,76] and later experimental studies, which show that these materials have finite elastic moduli.[77] Within a clay sheet, we have defined bonds between adjacent CG particles, an in-plane angle term (which keeps the clay CG particles in their hexagonal configuration), and an out-of-plane angle term, which gives the clay sheet its rigidity. These bond and angle terms were matched to atomistic simulation data. There were no intra-sheet non-bonded interactions, as the out-of-plane angle term has sufficient rigidity such that the clay sheet is unable to roll-up or crumple and interact with itself. Non-bonded interactions were present between a CG clay particle and CG clay particles on other sheets.

The CG interaction potentials have a cutoff of 20 Å and are stored as look-up tables. The maximum timestep used was 8 fs, although the majority of the simulations used a 4 fs timestep for numerical stability reasons. The initial structures were created using a Monte Carlo procedure to produce a low energy state, drawing on the methods of Theodorou and Suter[78] and the look-ahead procedure developed by Meirovitch.[79] A look-ahead of 4 bonds was considered. The details are as follows:

1. The clay tactoid is inserted in the center of the simulation box. For each polymer molecule to be inserted, 10 trial insertions of the first CG unit are performed, calculating the Boltzmann factors for each trial insertion.
2. One insertion is selected according to its Boltzmann weighted probability. If the energy of all the insertions is too high (which we define as the sum of the Boltzmann factors being less than 1 × 10^−6^), the process is repeated until a successful low-energy insertion has been made.
3. When the insertion of all the first CG units is completed, the rest of the polymer chain is created by adding each CG unit in turn for all polymer molecules.
4. We then randomly select the bonds, angles, and dihedrals for the next 4 CG units in the chain using a probability based on their potential forms. Here, 32 look-ahead trial positions are generated for the addition of each polymer CG unit.
5. Once these 4 CG particles are added, we calculate the non-bonded interaction energies using a cutoff of 20 Å and compute the Boltzmann factors for each of the 32 trial configurations from the interaction energies. Again, if the Boltzmann factor total is below the threshold (set at 1 × 10^−6^), we categorize the additions to be of high energy and create 32 new trial growths. If this repetition occurs more than 3 times (i.e., 128 trial set generations have been produced without meeting the threshold), then the most recently added CG unit is removed and the procedure restarts (so-called “recoil growth”). If the previous CG unit added is the first CG unit, then we attempt to reinsert the first CG unit into the system.
6. If the sum of Boltzmann factors is sufficiently high (>1 × 10^−6^), we select a trial configuration according to its Boltzmann weighted probability and add the next CG unit to the chain. We repeat this procedure until all CG units have been added, thereby generating an amorphous polymer system in a relatively low energy state and avoiding high-energy overlaps. This structure is subsequently energy minimized using a steepest decent algorithm before being used in the CG molecular-dynamics simulations.

### 4.4. Deriving System and Materials Properties

To determine the number of polymers to insert in the system, we used the density from the simulations of pure atomistic poly­mer at a pressure of 1 atmosphere and a temperature of 500 K. To calculate the volume in the simulation cell occupied by the clay sheets, we applied a grid of spacing 1 Å to the simulation box, and calculated the sum of grid points within a distance of 6.5 Å from the clay CG platelet (the value at which the interparticle clay–polymer potential is close to zero). We used a density of 0.772 g mL^−1^ for the PEG systems and 0.753 g mL^−1^ for the PVA systems. The elapsed simulation times reported in this article are the effective simulation times, estimated by comparing polymer diffusion coefficients at the atomistic and coarse-grained level. Based on this comparison, the effective simulation time is 6.5 times longer than the time obtained directly from the CG simulation output.

To compute the elastic properties of the “long”-PVA and “long”-PEG nanocomposite systems, we performed uniaxial tension and compression simulations using our CG models. This allowed us to determine the stress–strain behavior and to observe the role of the clay platelets during the deformation mechanisms. These simulations were purposely performed at a low temperature (100 K), below the glass-transition temperature for all the polymer systems, as evidenced by the initial elastic regime observed at small strain values. At higher temperatures (300 K and 500 K) the stress–strain curve does not show an elastic regime, as expected for polymers above the glass-transition temperature or as a melt.

**Figure 3:**
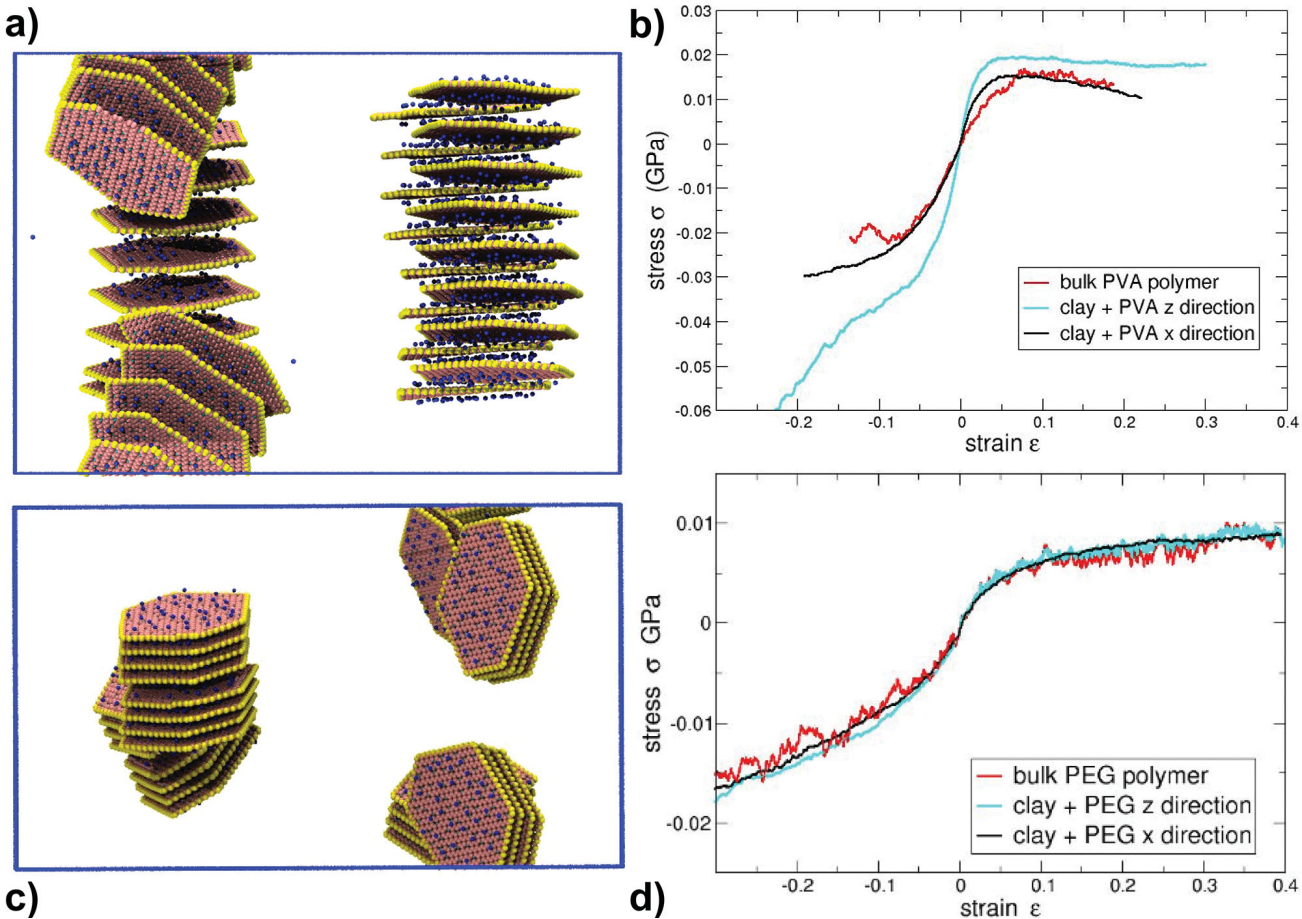
a) The initial structure used in the uniaxial tension and compression calculations for the “long” PVA system displayed; The *x* direction is along the horizontal axis, and *z* direction along the vertical axis. Note that the platelets are mainly orientated in the *xy* plane. b) The corresponding stress–strain curves in the *x* (black) and *z* directions (cyan) directions for the system shown in (a). The neat polymer stress–strain curve is also shown (red). c) The initial structure for the uniaxial tension and compression calculations for the “long” PEG nanocomposite, also shown along the x direction (horizontal axis) and the *z* direction (vertical axis). d) The stress–strain curves for the “long” PEG nanocomposite shown in (c).

To create the initial conditions for the uniaxial simulations, the final positions from our models with “long” polymer chains at 500 K were cooled down to 100 K over 4 ns and subsequently simulated at 100 K for 2 ns, with no drift in the lattice parameters observed at the end of the simulation. An illustration of the initial conditions for the long-PVA clay nanocomposite system is shown in **Figure** **3**a. The CG nanocomposite systems were deformed in the *x* and *z* directions under a uniaxial tensile strain at a constant strain rate (1 × 10^−8^ s^−1^) with a zero pressure condition imposed on the lateral faces. The stress components were determined from the pressure tensor, calculated via the virial stress. For comparison, a bulk polymer system was simulated at 500 K and subsequently cooled to 100 K. This was subjected to the same stress–strain regime as the nanocomposites. Note that the calculations reported for the studies at 100 K are not expected to be quantitatively correct, as they employ potential parameterizations obtained at higher temperatures (both those from the all-atom ClayFF and the CG parameterization).

### 4.5. Computational Techniques

**Figure 4:**
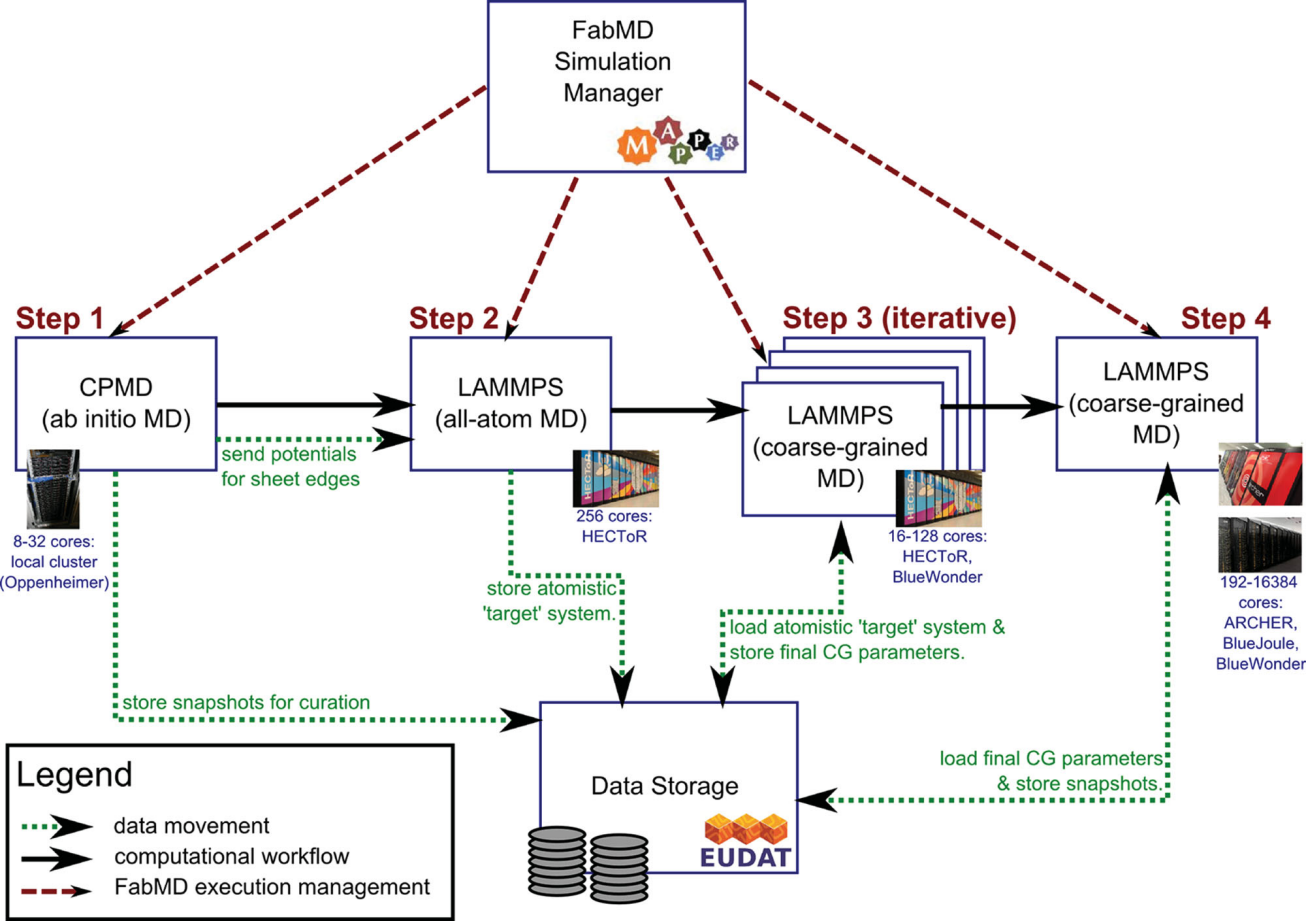
Overview of the computational and data workflow applied in our multiscale modeling scheme. We use the FabMD toolkit to help automate the four primary steps in our approach. These include ab initio calculations of the forces on the sheet edges (step 1, run on a departmental cluster), all-atom MD simulations to extract key distribution functions (step 2, run on a supercomputer), hundreds of small coarse-grained MD simulations performed iteratively to find the optimal parameters for the coarse-grained system (step 3, mostly run on a supercomputer), and finally the coarse-grained MD production runs (step 4, with simulations run on several different supercomputers). For each step in the workflow, we provide basic information about the HPC resources used below the description box. We use a distributed data-storage system, provided by EUDAT (http://www.eudat.eu), to facilitate the exchange of data between the different steps of the workflow and to curate the output of our simulations.

We summarize the computational workflow underpinning our calculations in **Figure** **4**. The workflow involves four steps; respectively, to determine the charges on the quantum mechanical level (step 1), to calculate accurate radial distribution functions and forces on the atomistic level (step 2), to iteratively assess the properties of trial coarse-grained configurations (step 3), and finally to run coarse-grained production simulations at full scale (step 4).

From a computational perspective, step 3 of the workflow is particularly extensive, as it involves applying IBI to obtain the polymer–polymer interaction potentials and calculating the potentials of mean force to obtain the polymer–clay and clay–clay potentials. Overall, this requires the execution of 5 to 30 simulations per interaction potential for IBI (each simulation using 16–128 cores), and 60 to 80 small simulations per interaction potential for calculating the potential of mean force (each simulation using a single core). In total, approximately 1200 simulations are needed to resolve an individual system, ranging from parameterization runs that require processing on a single core for a minute, to large production runs that require processing of thousands of cores for a week or more.

A wide range of computer platforms were used in this work to match the computational requirements of the particular simulations being performed. For the Car–Parinello MD (CPMD) DFT simulations,[80] we used a departmental Intel SandyBridge cluster with Infiniband (named Oppenheimer), while the LAMMPS[68] classical MD simulations ran variously on lower- and higher-end supercomputers, as part of PL-Grid (AMD Opteron-based machines, see http://www.plgrid.pl), BlueJoule and BlueWonder (an IBM BlueGene/Q and an iDataPlex machine at Hartree Centre, DL), HECToR and ARCHER (Cray XE6 and XC30 machines at EPCC, Edinburgh), and JUGENE (an IBM BlueGene/P supercomputer at FZJ, Juelich).

Coupling ab initio, atomistic and coarse-grained MD models is a challenge that has been tackled using diverse approaches.[81] These include, for example, the use of bespoke scripts, which are effective to quickly address scientific challenges in coupling models for a specific problem, but are limited in reusability and flexibility when compared to publicly released software packages. As a result, a number of general packages have been released for coarse-graining (e.g., VOTCA[82] and MagiC[83]) and for coupling models in general (e.g., MUSCLE[84] and FeniCS.[85]) These approaches are flexible, but require considerable domain and technical expertise to solve specific multiscale science problems. To make rapid progress in the field of multiscale modeling, we require toolkits that are flexible and generalized, that can conveniently make use of high performance computing resources, and that can also be systematically applied to specific problems.[86] Fortunately, several of these approaches are now appearing in different scientific disciplines.[43,81]

In our work, we chose to combine general tools with bespoke scripts, and to evolve scripts gradually into more general tools in cases where we frequently relied on their functionalities. Our general tools were developed in the MAPPER project[87–90] to make this set of calculations manageable, and to introduce automation in the various steps of our multiscale scheme. Most notably, we have developed a Python-based toolkit, named FabMD, which allows us to fully automate iterative Boltzmann inversions for individual potentials, combining local testing and analysis procedures with the configuration and execution of simulations on various remote supercomputers. The use of multiple resources is beneficial to us for several reasons: i) to map simulations to resources that are well suited for their computational and storage requirements; ii) to sustain the workflow in cases when one or more resources are undergoing maintenance or become heavily oversubscribed.

The FabMD toolkit, and its general-purpose equivalent FabSim (http://www.github.com/djgroen/FabSim) were originally derived from a Python package named Fabric,[91] itself developed to aid in the administration of computer systems. The automation offered in this context is also of considerable benefit in the implementation of our scheme. Indeed, we have greatly extended the original framework, and developed FabMD in lock-step with this project (see Supporting Information). We intend to package and publicly release the toolkit in the near future.

On aggregate, our distributed simulations generated approximately 5 TB of simulation data. The storage for this data has been provisioned in a distributed data-management system (EUDAT: http://www.eudat.eu).

## 5. Intercalation

We observe the process of polymer melt intercalation into the galleries, formed by the nanometer wide spacing between each clay sheet (or “platelet”) within the initially unintercalated tactoids, which comprise the starting structures in our models. The process of intercalation is a fundamental mechanism through which molecules and ions enter and leave these materials by adsorption and desorption. However, until now, this has been out of reach of either experimental observation or computational simulation. Experimentally, the processes are too rapid for even the most-sophisticated experimental techniques available today to be able to resolve molecular details within the galleries. In addition, clays are often poorly characterized (disordered) materials. As almost all simulations of these materials have been based on models with periodic boundary conditions imposed on the clay sheets themselves, it has been impossible to observe these intercalation processes. Because of the chemically specific bridging of length and time scales employed in this paper, we are able to describe not just a pair of finite and adjacent clay sheets and their associated gallery, but substantial numbers of tactoids complete with edges, embedded in a molten polymer sea. Moreover, as our earlier very large scale all-atom simulations of periodic (cationic and anionic) clay sheets demonstrate,[63,92] they are flexible and have elastic properties including bending and Young's moduli. Indeed, the intercalation and anomalously high diffusion coefficient of intercalated nucleic acid polymers was shown to be due to the flexibility of the clay sheets, a peristaltic mechanism being proposed to account for the motion of these polyanions within galleries.[93] We provide visualizations of two exemplar intercalation processes in Figure 2.

At 500 K, we observe rapid intercalation of “small” and “long” PVA polymers into the galleries within a few tens of nanoseconds, with most of the intercalation complete after 100 ns (albeit in some cases intercalation continues for a further 400 ns). What is particularly evident is that the galleries into which the PVA-mers first intercalate are those for which fluctuations in the clay sheet, due to its elastic properties, cause the edge of the gallery to widen significantly beyond the notional average *d*-spacing. Once one PVA-mer has entered, attracted at least in part by the presence of the intercalated Na^+^ species, it entrains an inward flux of further molten polymer molecules, which advance en masse into the gallery, driving a “solitary wave” of increased interlayer spacing which propagates along the pliable aluminosilicate sheets. This behavior corresponds globally to theoretical predictions made by Ginzburg et al.*,*[94] based on a continuum model that supports a Korteweg–de Vries soliton wave. See Figure 2a–c for a dramatic illustration of this mechanism, and Figure 2g–h for the corresponding sheet angle and *d*-spacing measurements. Fu et al.[56] have shown that montmorillonite clay sheets can absorb large energies before fracture; here, we observe the bending to be well within their observed failure bending radius limit of 20–30 Å. The equivalent PEG-mers show no propensity to intercalate, due (we infer) to the lack of sufficient attractive forces between these polymer molecules, the clay sheets, and the sodium ions in the interlayer region.

Overall, for the clay–PVA system, we measure an average nearest-neighbor distance of about 22 Å between adjacent clay sheets when these are intercalated with PVA. The polymer adopts a pseudo-trilayer arrangement in the interlayer gallery. This is in accordance with observations from the experimental literature,[95] which report *d*-spacing distances ranging from 20 to 30 Å. The final configuration of systems containing PEG in our simulations depends strongly on the initial conditions. In the runs where we commence with 8 tactoids of unintercalated clay sheets, we find that no intercalation occurs within the first 1.3 μs. In contrast, if the initial configuration comprises dispersed clay platelets, the sheets rapidly assemble into tactoids, incorporating a monolayer of PEG between the clay sheets. After 1.95 μs, we observe an average *d*-spacing of about 16 Å. The initial and final structures formed by the exfoliated “long” PEG–clay tactoid are shown in Figure 2. Based on these results, we conclude that the PEG system is more likely to reach intercalation when processing conditions are such that the clay itself is initiated in a more-dispersed state, or when much more force is applied so as to induce the PEG to intercalate into the tactoids. Intercalation of PEG with MMT, subject to external pressure, has been experimentally reported by Chen and Evans.[96]

**Figure 5:**
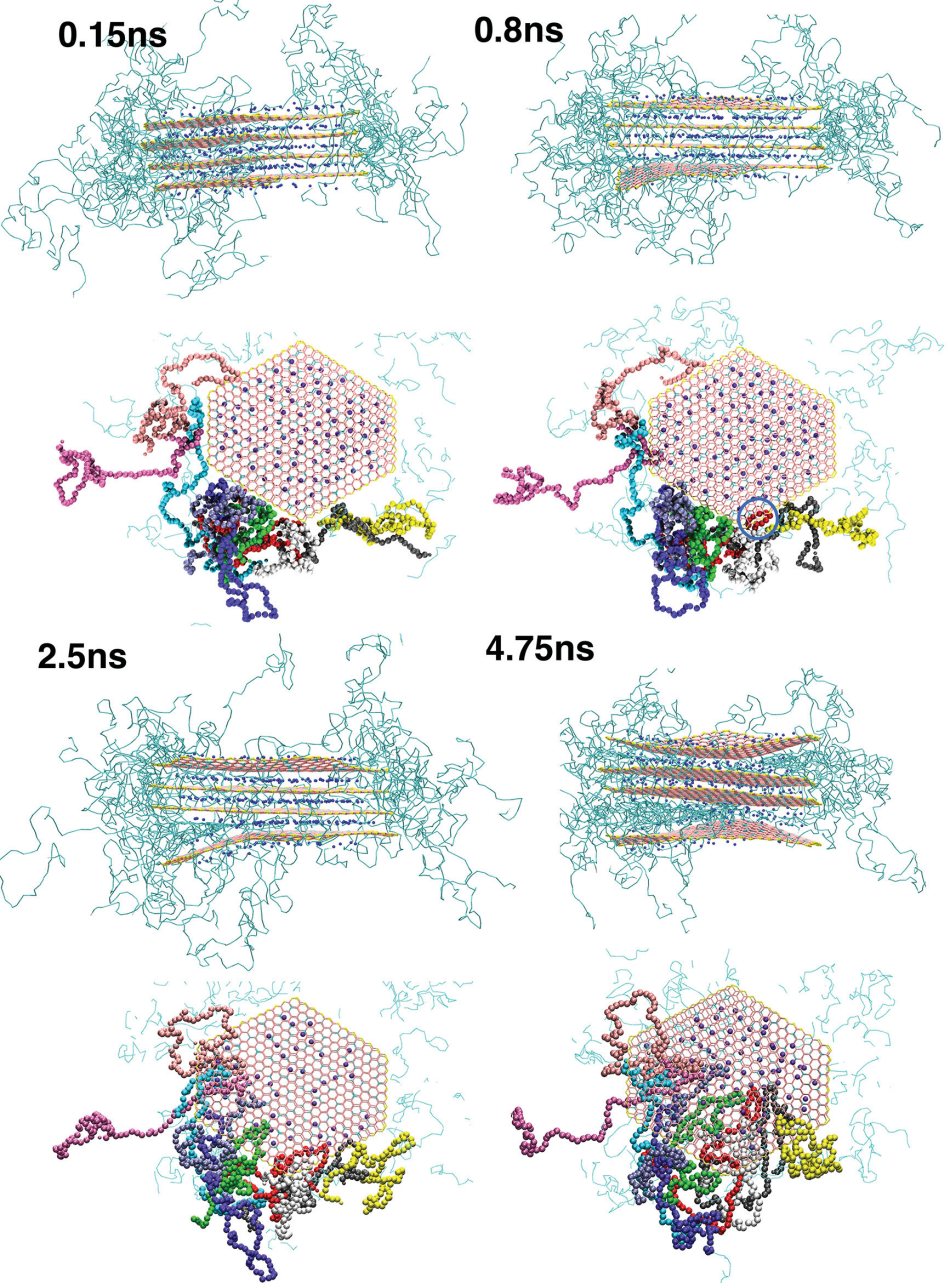
Pictorial overview of the intercalation of “long” PVA polymers into a selected clay tactoid. We illustrate the side and top views of the tactoid at relevant snapshots, for the simulation times shown. In the side views, the colours of the CG particles are the same as in Figure 2. For each timeframe, the side view illustrates the bending that the lowermost clay sheet undergoes to accommodate the intercalating PVA polymer molecules. For the top view, the polymers that intercalate into the spacing between the lowermost sheets are coloured according to their molecule number, such that they can be differentiated during visualization. We observe the polymer initially intercalating as short loops (an example is circled in blue at the 0.8 ns snapshot), which progress further into the interlayer and form a relatively linear chain on the clay surface.

**Figure 6:**
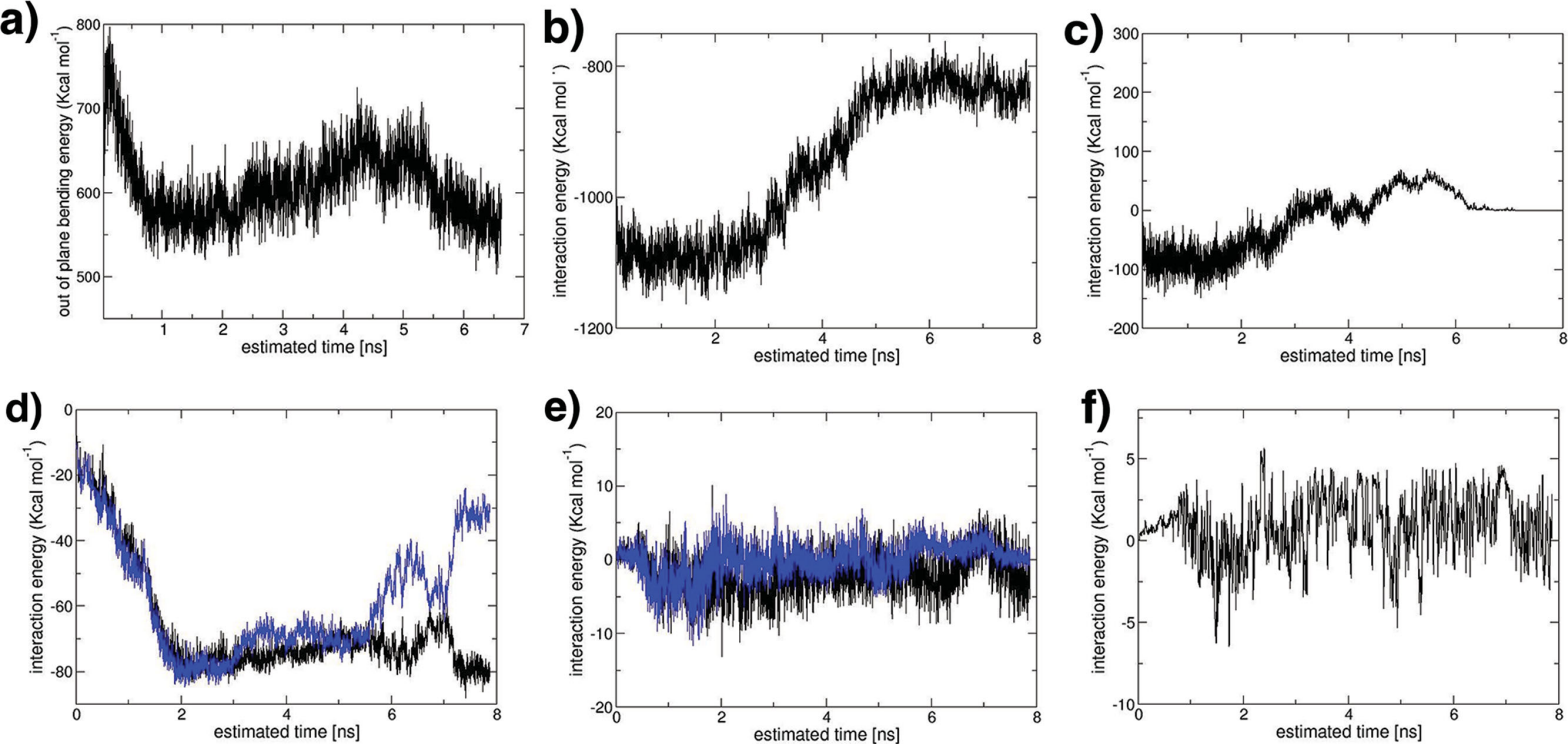
Energetic contributions during the intercalation process illustrated in Figure 5. a) The out-of-plane bending energy of the lowest clay sheet shown in Figure 5; the bending energy peaks at 0.15 ns before reaching a minimum at 0.8 ns. As the solitary wave propagates across the clay sheet, the bending energies become higher as the curvature becomes greater. Once the whole interlayer is intercalated (7 ns) the clay sheet is predominately flat and the bending energies are at their lowest value. b) The interaction between sodium CG ions and the lowest clay sheet. The energy increases as the clay sheets separate after 2 ns. c) The interaction energy between the lowest and the adjacent clay sheet. This becomes less favourable as intercalation progresses. d) The interaction energies between the first five CG intercalating units of the representative PVA molecule illustrated in Figure 5b and the clay neutral CG particle of the lowest clay sheet (black line) and the adjacent clay sheet (blue line). The interaction energies become significantly more favourable as the PVA molecule intercalates. e) The interaction energies between the first five PVA CG intercalating units and the clay charged CG particles of the lowest clay sheet (black line) and the adjacent clay sheet (blue line). As the polymer initially intercalates, the charged-clay–PVA interactions are favourable. However, after 2 ns, the sodium ions migrate to the sites of the charged clay particles, and the favourable interactions are reduced. f) The interaction energies between the first five CG intercalating units of the representative PVA molecule and sodium ions.

We have further determined the driving force for intercalation by analyzing the energetic changes during the initial stages of polymer intercalation into a clay tactoid, shown in **Figure** **5**. In the example we have chosen, the “long” PVA polymer molecules initially intercalate into the outer sheets of the four sheet tactoid. After only 5 ns, we find intercalation is almost complete into these interlayers. The expansion of the clay interlayer spacing to accommodate the polymer has two energetic consequences: i) the sodium ions interact more weakly with the clay sheets, as the sodium ions migrate onto a single sheet and are no longer equidistant between the two sheets; and ii) the energetically favorable interactions between clay sheets decrease as the sheets move apart. We show the energetic changes for the outer clay sheet during the intercalation process in **Figure** **6**b,c) for these two processes, respectively. In the case of PVA molecules, the interaction of the polymer molecules with the large number of coarse-grained clay sheet particles (both charged and neutral) is enough to overcome these energy losses (and any entropic penalty due to confinement), as shown by the intercalated structure being the most-stable one observed in our simulations.

**Figure 7:**
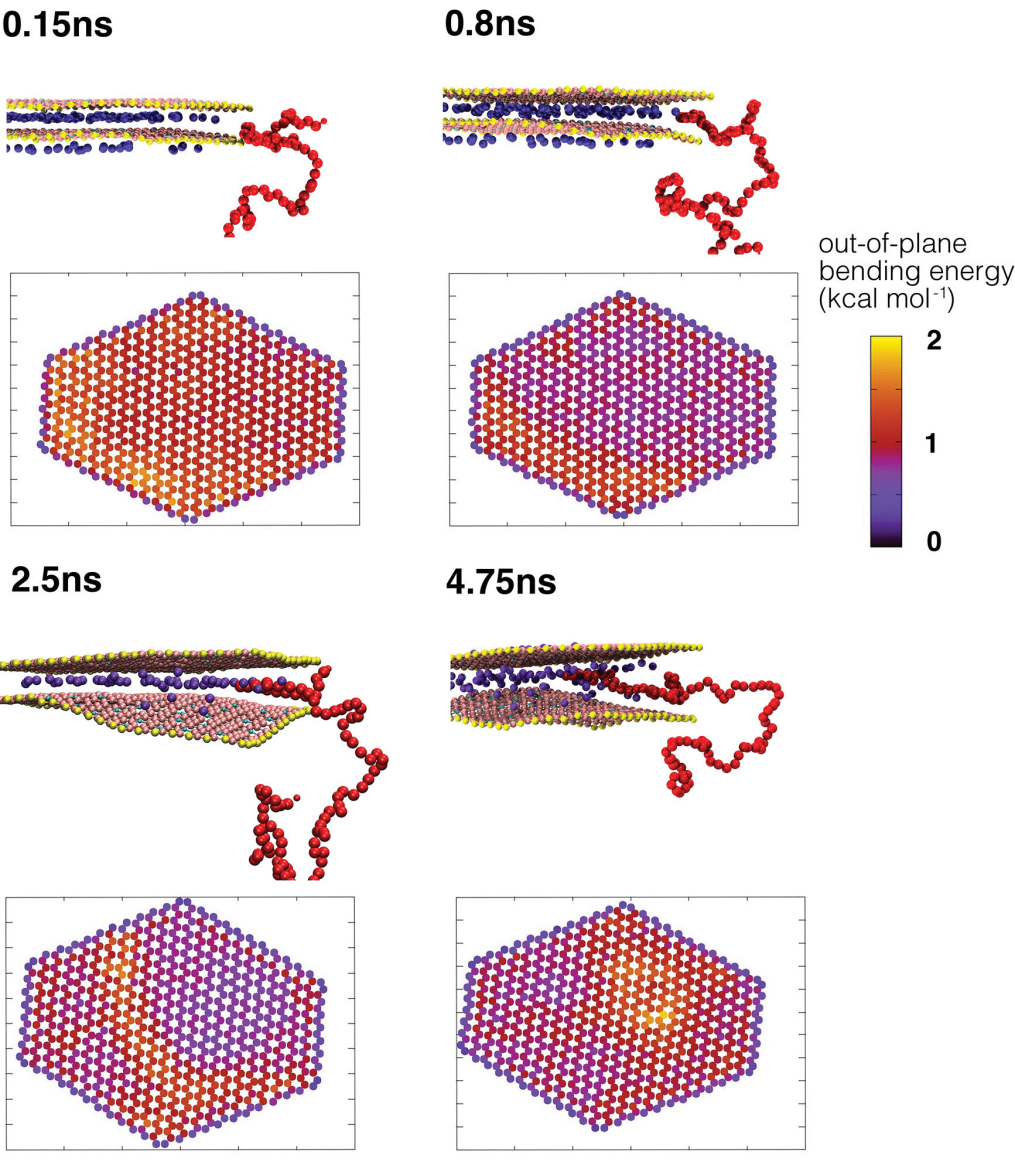
Detailed pictures of the intercalation of “long” PVA polymers: for each snapshot; we show the side view of the edge of the clay platelet with the intercalation of a representative polymer molecule (the red polymer molecule in Figure 5). We also show the out-of-plane bending energies as a colour map for each coarse-grained clay particle in the lowest clay sheet in Figure 5, averaged over 100 snapshots (approximately 0.01 ns), the position of the clay particle being projected onto the *xy* plane. The sum of these bending energies is shown in Figure 6a. These energies are initially high near the point of intercalation at 0.15 ns, and by 0.8 ns have coalesced into the start of the solitary wave that we observe during intercalation. This causes the entrance to the interlayer gallery to expand, and the red molecule to intercalate, as shown in the side view. By 2.5 ns, the solitary wave and the associated high bending energies have progressed along the clay and the polymer molecules have intercalated into the centre of the clay. By 4.75 ns, the only region left unintercalated has a high bending energy region around it; the now intercalated parts of the clay sheet are predominately flat.

However, the initial intercalation process requires the clay sheet to bend to increase the interlayer spacing in the region of intercalation; the bending of the clay sheets requires additional energy. This is shown in 6Figure a, where we have summed the out-of-plane bending energy of the outer sheet particles during intercalation. As Fu et al. have illustrated both theoretically and through TEM images, clay sheets can possess very large curvatures without failure.[11] The out-of-plane bending energies quickly increase to a maximum after approximately 0.15 ns. In Figure 6c, we illustrate the location of the highest bending energies on the clay sheet; we find these are located where the polymer molecules subsequently intercalate. By 0.8 ns, the numerous high-energy, small-amplitude bends have coalesced into the start of the solitary wave we observed during intercalation, illustrated by a high-energy out-of-plane bending line which propagates across the clay sheet in **Figure** **7**. As a consequence, by 0.8 ns, the total out-of-plane bending energy is much reduced. At this point, the first polymer molecules have penetrated into the clay interlayer spacing. Most polymer molecules initially enter the spacing in a loop consisting of approximately three to five CG particles. We use molecules colored red in Figure 7 to illustrate this process and, in Figure 6, we show the energetic changes as these five CG particles enter the clay galleries. Initially, the polymer molecule is resident on the clay edge; the favorable interactions with the clay sheet (both neutral and charged) within the interlayer are such that the molecule is drawn towards the interlayer, causing the entrance to the gallery to expand (and the associated bending energy of the clay sheet to increase). By 0.85 ns, these polymer CG particles are now fully within the interlayer spacing and experience many favorable interactions with the clay sheet (Figure 6d,e). However, for the polymer to further ingress, the sodium ions represent a steric hindrance. We have observed that, on some occasions, the sodium ions jump-diffuse to other charge sites but, by 2 ns, a transition occurs wherein the sodium ions start to shift onto one of the two clay sheets as the interlayer spacing increases and the polymers intercalate further (Figure 6b), a process determined by the free energy of cleaving the clay surface.[5] Once this process has started, the polymer intercalates around, above, and below the sodium ions. When a polymer molecule is close to a sodium ion, this leads to favorable interactions as shown in Figure 6f. However, it is the favorable interaction between the PVA polymer and the clay surface that drives intercalation, compensating for the energetic penalty due to sodium-ion migration and the clay sheets moving apart; it is also sufficient to overcome the barrier to clay-sheet bending. In no cases do we see the initialization of intercalation followed by the polymer retreating from the interlayer with the clay interlayer returning to its unexpanded state; once the intercalation has been initiated, the process is irreversible.

**Figure 8:**
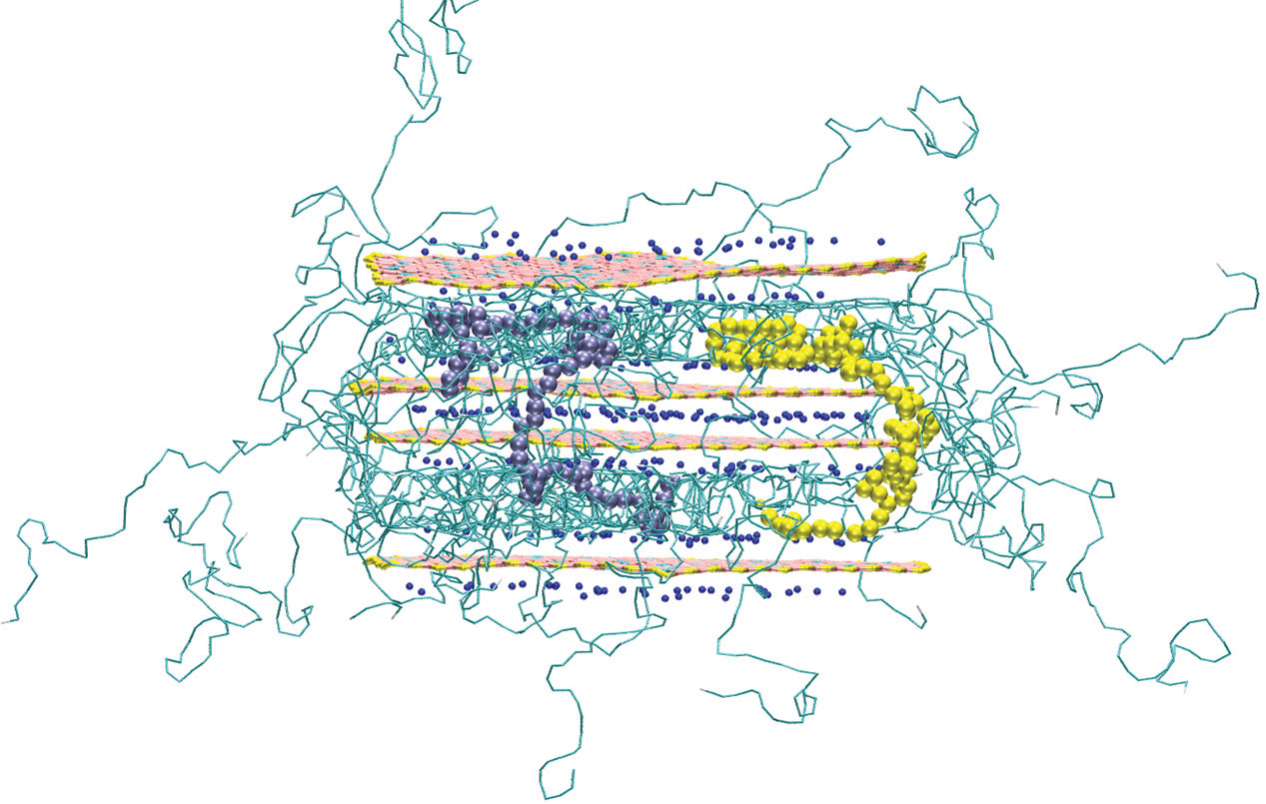
Visualization of the “long”-PVA clay system described in Figure 5 after 7.5 ns. We have highlighted two molecules, coloured in purple and yellow, which are both simultaneously intercalated in two different interlayer spacings. They act as a barrier to intercalation into the middle interlayer spacing, which remains unintercalated throughout the simulation.

During intercalation, we observe the molecules diffusing toward the centre of the clay sheet in a relatively linear fashion. As the head group of approximately three to five polymer CG particles moves further into the interlayer, the chains behind flatten and adopt a more-linear confirmation. We illustrate this configuration with different-colored molecules in the final snapshot in 5Figure. With the “long” polymer in its stretched form (rather than the coiled form seen in the bulk), the end-to-end distance of the polymer is now more than the diameter of the clay sheet. As a consequence, a large fraction of intercalated polymer molecules still resides outside the interlayer spacing. In many cases, we observe the non-intercalated end of the polymer intercalating into another interlayer spacing within the tactoid; we illustrate this in **Figure** **8**, where polymer molecules bridge between the first and the third interlayer spacing. This provides a barrier to intercalation into the middle spacing, which remains unintercalated through the remainder of the simulation.

**Figure 9:**
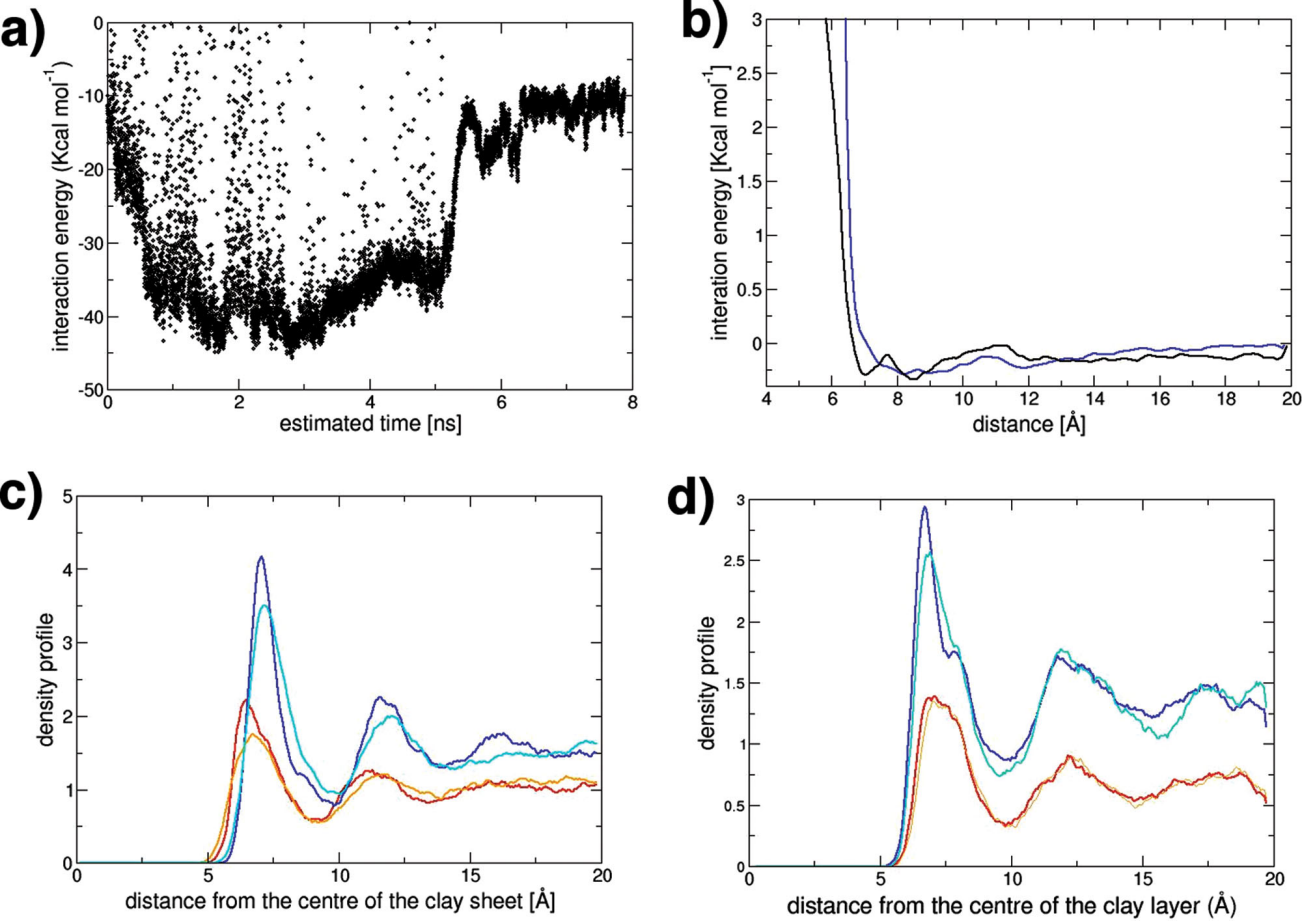
a) The interaction energy of the first five intercalating CG units of the red molecule illustrated in Figure 5b with neutral clay particles, using the PEG CG interaction potentials. The corresponding energies for the PVA system are shown in Figure 6d. We see that the interaction energies are noticeably less favourable for the PEG–clay system, with some very high energy configurations, compared with the PVA–clay equivalent. b) The CG interaction potentials between the CG neutral clay particles and the CG polymer monomer unit for PVA (black) and PEG (blue). The PVA interaction parameter is more negative at shorter distances than the PEG potential; this is the reason for the more favourable interaction energies of the PVA–clay system. c,d) The density profile perpendicular to the clay surface for the uncharged clay and the PEG polymer and the PVA polymer respectively. The lines are coloured as follows: blue is the density profile for the monomer units calculated from an atomistic simulation, cyan is the density profile for the corresponding CG simulation, red is the density profile for the terminal units calculated from an atomic simulation and orange is the density profile from the corresponding CG simulation. We see that the PVA–clay system is approximately 0.5 Å closer to the clay surface, due to the PVA molecules' greater attraction for the clay surface.

As noted above, for the PEG–clay simulations, we observe no intercalation, although we do observe intercalated structures when the starting structure is exfoliated. To understand the energetic reasons for this, we have taken the same intercalation snapshots from our “long”-PVA simulation, and replaced the non-bonded interaction potentials with those derived for the PEG–clay system. In **Figure** **9**a, we show the interaction energy between the PEG 5 CG-particle head group and neutral clay-sheet particles. We notice that, although favorable, the energies are less than those for the corresponding PVA–clay system (Figure 6d). There are also some very high energy regions, as the PEG–clay distance is within the repulsive, short-distance part of the interaction potential, shown in Figure 9b. Both these observations can be understood in terms of the polymers interacting with the clay surface. The density profiles perpendicular to the clay surface for a periodic system of uncharged clay and polymer are shown in Figure 9c,d for the clay–PEG and the clay–PVA cases respectively. The monomer units of the PEG are found to be 7.15 Å from the clay surface, while the equivalent PVA monomer units reside at 6.65 Å; the monomer units in the latter are closer due to the hydrogen bonding interactions of the hydroxyl groups of the PVA with the silicate oxygen atoms of the clay surface. As a consequence, the CG interaction potential for clay–PVA is more negative at shorter distances and does not become repulsive until significantly shorter distances. The outcome is that the PEG–clay interaction is insufficiently favorable to overcome the bending-energy requirements of the clay sheet for intercalation to occur, which are also likely to be higher as the PEG CG units are larger, and the spacing required to initiate intercalation greater.

## 6. Self-Assembly of Tactoids

**Figure 10:**
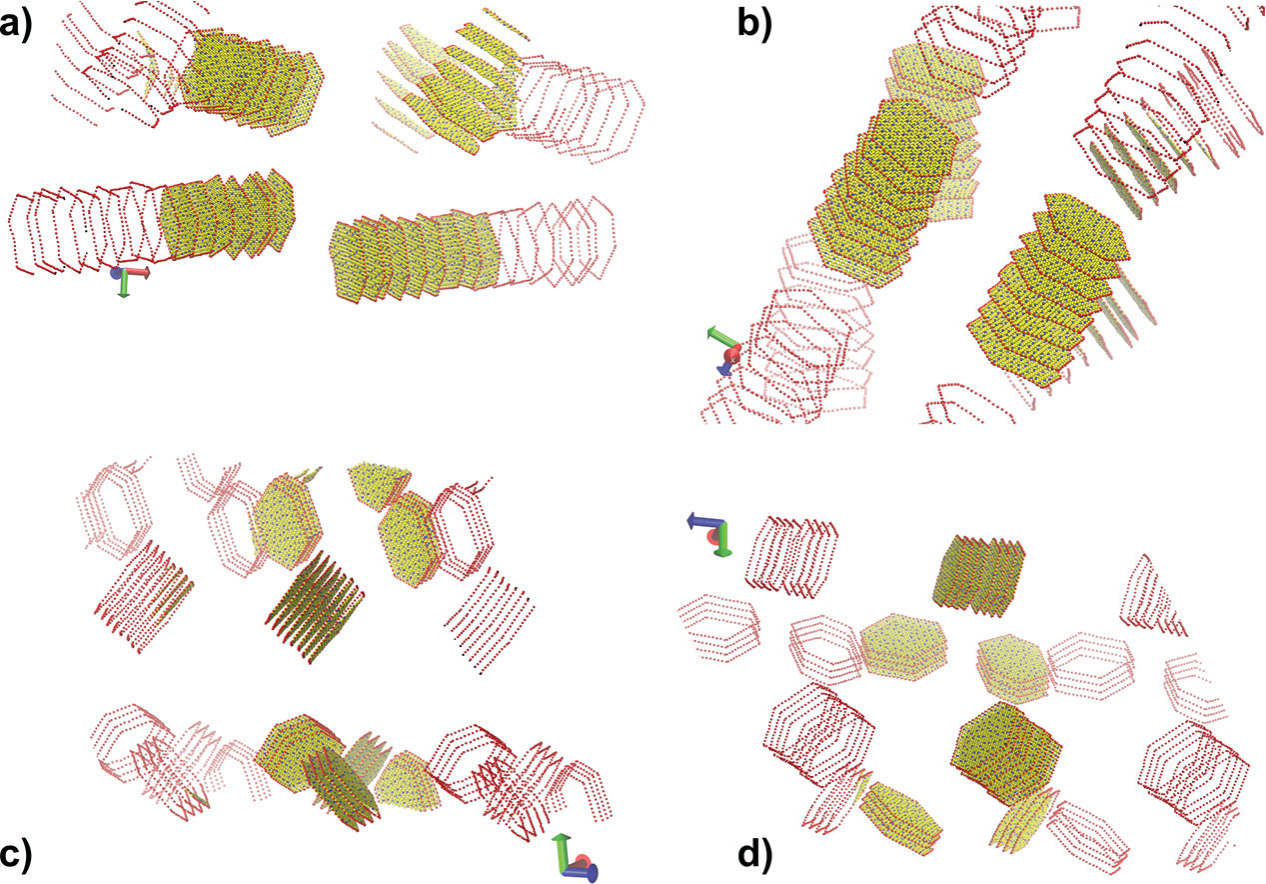
We show the clay platelet arrangements for the PVA–MMT system with: a) 31–35 monomers per PVA molecule after 1 μs, and b) 100 monomers per molecule after 0.5 μs; and for the PEG–MMT system with: c) 31–35 monomers per PEG molecule after 1.3 μs and d) 100 monomers per molecule after 1.7 μs. We show all the platelet particles that reside within the simulation volume, including the uncharged sheet particles (in yellow), the charged sheet particles (blue), and highlight the particles on the sheet edges in red. We display the edges of selected platelets beyond the periodic boundary, but none of the polymers, to aid the visual recognition of larger-scale structures.

The interaction between the clay sheets and the polymer matrix determines the unusual materials properties of these nanocomposites.[97] The large space and timescales required to model these interactions has kept the study of these systems out of reach until now. We provide the final snapshots of clay tactoid self-assembly into larger structures in **Figure** **10**. Tactoids proceed to form increasingly organized clay–polymer structures in all our simulations during the first 100 ns, but these structures differ between the (intercalated) PVA and (unintercalated) PEG systems. Most of the platelet stacks consist of face–face arrangements in the two PVA systems, with only one face–edge arrangement and no edge–edge arrangements observed. In contrast, the PEG systems feature several edge–edge and a much smaller number of face–face arrangements. Given that both systems were initiated with an identical distribution of clay tactoids, we infer that the type of polymer included in these systems has a non-negligible effect on the self-assembly behavior of the clay tactoids, and through that on the materials properties of the resulting nanocomposite (see below).

**Figure 11:**
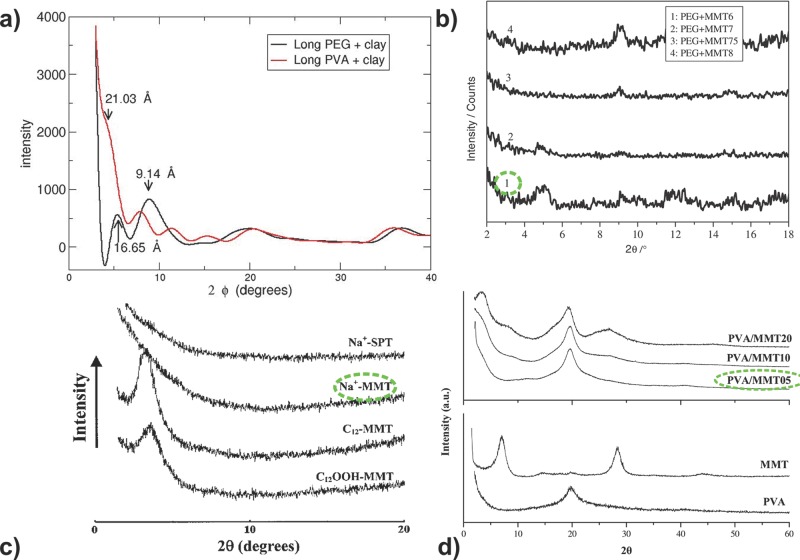
a) Computed X-ray diffraction patterns of the long PVA–MMT system and the long PEG–MMT systems at the final state of the simulations (respectively after 0.65 and 1.7 μs). We indicate the *d*-spacings corresponding to two of the peaks. b) XRDs of PEG–MMT with different volume fractions of PEG as reported by Chen and Evans.[100] (Reproduced with permission.[100] Copyright 2005, Taylor and Francis). The composite with 6% PEG is closest to the PEG–MMT systems we have modelled (see the line labelled “1” in the graph). c) XRDs of PVA–MMT as reported by Chang et al.[98] (Reproduced with permission.[98] Copyright 2003, Wiley). The sodium-charged MMT-based system is the closest to that which we have simulated (line labelled “Na^+^–MMT”). d) XRDs of PVA–MMT as reported by Sapalidis et al.[99] (Reproduced with permission.[99] Copyright 2011, The Authors, published by InTech, Rijeka, Croatia under a CC-BY-NC-SA 3.0 license; available from: http://dx.doi.org/10.5772/18217). The 5% PVA system is closest to that which we have simulated (line labelled “PVA–MMT05”).

Comparison with experimental data must proceed in general terms due to a lack of precision measurements on sufficiently well characterized experimental clay–polymer systems. We present a comparison of our computed X-ray diffractograms (XRDs) for the “long” polymer clay systems, along with the associated *d*-spacings, with those from literature sources in **Figure** **11**. The X-ray diffraction observations of MMT-Na^+^/PVA nanocomposites as obtained by Chang et al.[98] are broadly in agreement with our results; in both cases we observe a high intensity at very low angles with a slightly elevated intensity for angles corresponding to *d*-spacings of approximately 20 Å. Our results are also in general agreement with the XRDs from Sapalidis et al.*,*[99] both having a peak in intensity at an angle of ca. 4/5°. Comparing the MMT-Na^+^/PEG X-ray diffraction pattern with those reported by Chen and Evans[100] for a variety of heat treated and dehydrated montmorillonite clays, we see a peak in both the simulated and experimental spectra at approximately 9°, corresponding to an unintercalated clay (for PEG+MMT8, a montmorillonite clay heat treated at 800 °C). In our calculated X-ray diffractogram of the “long” PEG–clay system, we find a peak at 5.32°, corresponding to 16.65 Å, which is due to the face–face interaction between the clay tactoids. A similar peak is seen in the experimental XRD for PEG+MMT6 (a montmorillonite clay heat treated at 600 °C); these peaks correspond to an intercalated PEG–MMT composite.

## 7. Material Properties

In Figure 3 we show the response of the “long” polymer–clay CG systems to uniaxial tension and compression when cooled below their glass-transition point, as well as with respect to the initial configurations of the clay platelets. For the “long” PVA polymer clay system, the majority of the clay platelets lie in the *xy* plane, which allows us to differentiate the in-plane and out-of-plane components of the response of the nanocomposite. In Figure 3b, we show the stress response to uniaxial tension/compression in the *x* and *z* directions. Similar to the neat polymer, the nanocomposite exhibits an elastic regime, a yield peak region and, finally, softening. In line with the general experimental trend of clays enhancing the Young's moduli of such composites,[101] we find an increase in the Young's modulus from approximately 0.372 GPa for neat polymer to 0.637 GPa and 0.955 GPa for clay–PVA under uniaxial tension in the *x* and *z* directions respectively (using a linear regression for strains of 0 to 1%). Under compression in the *z* direction at high strain values (−15%) we observe strain hardening that is not observed in the neat polymer, due to the system now containing an intercalated stack of platelets spanning the *z* dimension.

In Figure 3d, we also show the stress–strain response to uniaxial tension/compression for the “long” PEG polymer–clay system. The stress–strains values for the *x* and *z* directions and the neat polymer are very similar: 0.127 GPa for neat polymer as compared with 0.137 GPa and 0.142 GPa for the *x* and *z* directions of the polymer–clay system, respectively.

Our simulations reveal that the presence of the clay platelets, when intercalated, increases the elastic modulus of the nanocomposite significantly. These enhancement mechanisms depend on the orientation of the clay platelets; the elastic modulus in compression or tension orthogonal to the plane of the platelet is higher than that in plane. However, non-intercalated platelets, such as those seen in the “long” PEG polymer–clay system, do not enhance the Young's modulus. This is consistent with the general view that the improvement in mechanical properties is directly related to the degree of dispersion of the clay layers in the nanocomposite.[101]

**Figure 12:**
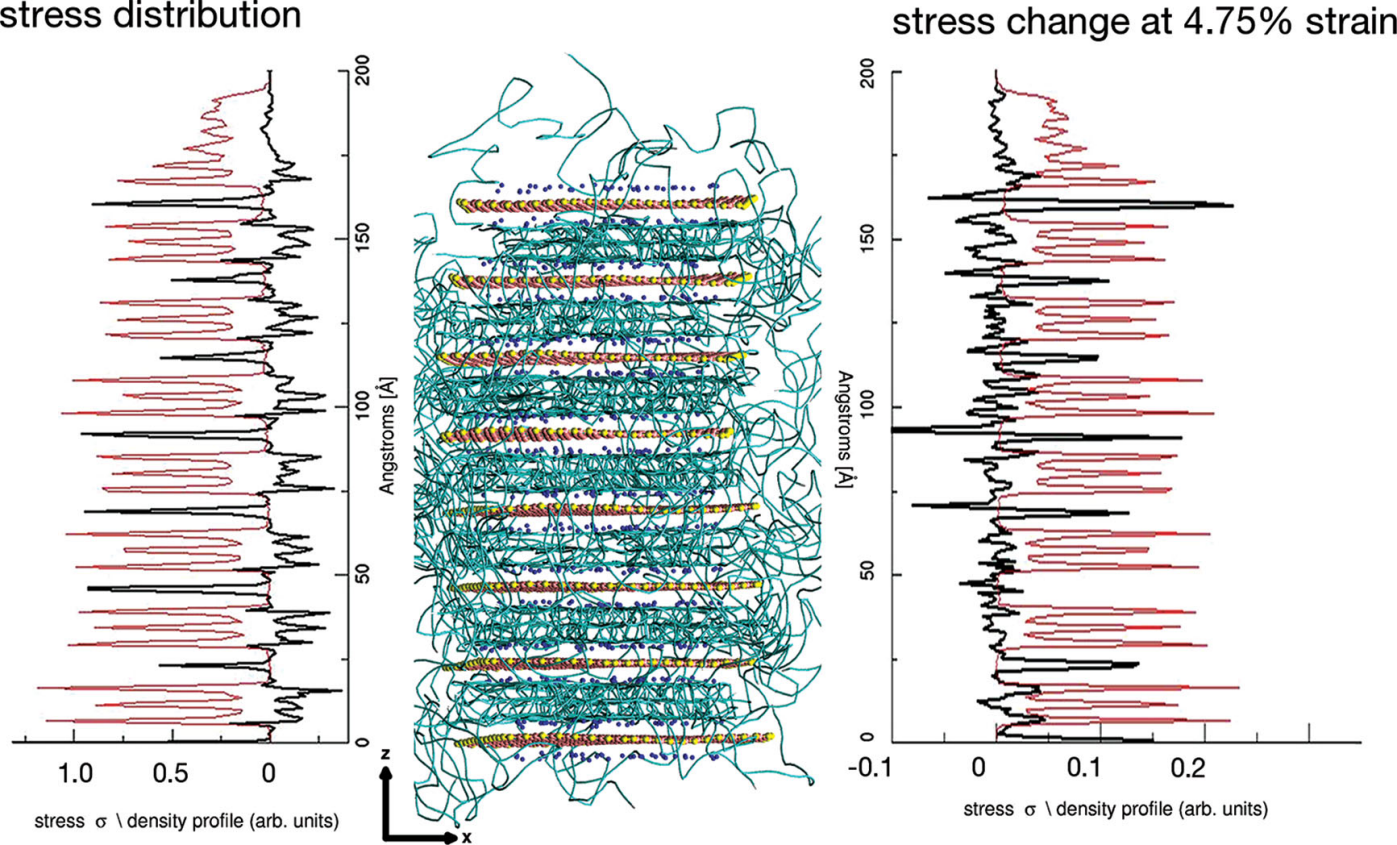
The stress distribution within a polymer-intercalated tactoid. Middle panel: the clay tactoid used to partition the Young's modulus. Each sheet in the tactoid lies approximately in the *xy* plane. The stack is approximately 166 Å high. The left panel shows the stress distribution (black line) for slices in the *xy* plane with a thickness of 0.75 Å. The stresses were calculated by summing the *σ_xx_* virial stress for each particle within the slice, and then dividing by the volume of the slice. The stresses shown are in GPa. The density profile of the polymer particles is shown as the red line. The clay possesses a positive stress, while the polymer stresses are negative, except for those molecules very close to the clay surface. In the right hand panel, we show the changes in *σ_xx_* when a uniaxial strain of 4.75% is imposed in the *x* direction (black line). We observe a positive stress response for the clay and in general, for regions of high polymer density, the change in stress is also positive.

To understand why this is the case, we have analyzed the deformation mechanisms of one of the tactoid stacks in our “long” PVA system when undergoing uniaxial strain in the *x* direction. This stack consists of eight platelets, lying in the *xy* plane; we provide an illustration in **Figure** **12**. We show both the *σ_xx_* stress distribution partitioned into slices in the *xy* plane with thicknesses of 0.75 Å in the *z* direction and the changes that occur in the *σ_xx_* stress distribution when a uniaxial strain in the *x* direction of 4.75% is imposed. The density profile of the polymer CG particles perpendicular to the *xy* plane is also shown; we find that regions of high polymer density have a negative stress, whereas near the clay surface, there are regions of positive stress. The clay sheets themselves possess a positive stress. This indicates that the majority of the polymer molecules are compressed in the interlayer, apart from those on the surface, which are stretched. Compressing the interlayer polymer molecules ensures as many polymer particles are within the interlayer as possible, whereas stretched polymers on the surface ensure that the particles are spread out on the clay surface. Both situations serve to enhance favorable clay–polymer interactions. When a strain of 4.75% is imposed, integrating across the tactoid, we find a positive *σ_xx_* response, as expected from the stress–stain curves presented in Figure 3. We find that the majority of the stress response stems from the clay sheets, indicating stress transfer from the polymer molecules onto the clay. The polymer molecules show a correlation between positive stress response and high polymer density, especially for polymer molecules close to the clay surface.

**Figure 13:**
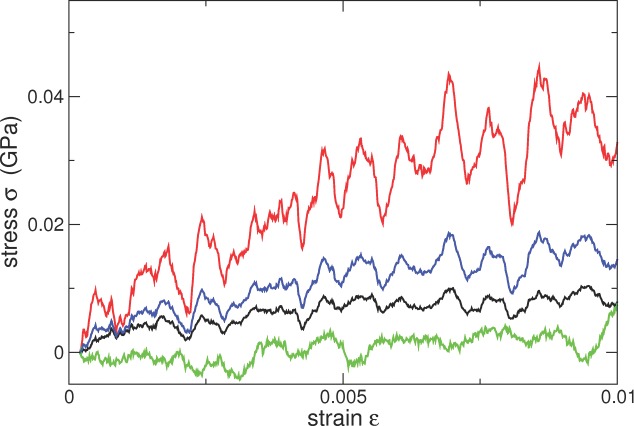
Stress–strain curve for uniaxial tension in the *x* direction, partitioned into the response from the clay, intercalated polymer and the tactoid as a whole, for the system shown in Figure 2. A) The stress (*σ_xx_*) is calculated as the sum of the virial stresses divided by the volume of the atoms in the group (calculated via a Voronoi tessellation). All the stress–strain curves are shown relative to their value at zero strain. The stress–strain response for non-intercalated polymer is shown in green. The red line, with the largest gradient (Young's modulus) is the stress–strain curve for the clay. The black line is the stress–strain curve for intercalated polymer molecules; we see that the gradient is less than for the clay but significantly higher than for the non-intercalated clay (0.68 ± 0.1 GPa versus 0.48 ± 0.1 GPa). The blue line is for the tactoid (clay + intercalated polymer) taken as a whole, i.e., an “effective particle”. We find that the Young's modulus of the whole tactoid is also much higher than that of the non-intercalated polymer at 0.42 ± 0.1 GPa.

We can now comprehend why the intercalated “long” PVA system has a much greater increase in Young's modulus than the equivalent “long” PEG system. The intercalated polymer molecules effectively transfer stress onto the much stiffer clay sheets, while the high-density intercalated polymer molecules also contribute to the increased Young's modulus. To demonstrate this, in **Figure** **13**, we show the stress–strain curve for the tactoid illustrated in Figure 2, partitioned into contributions from the clay sheet, the intercalated polymer, the tactoid as a whole, and, for comparison, a region of non-intercalated polymer. We see that the clay sheet has the largest gradient, demonstrating the transfer of stress from the polymer onto the clay. The intercalated polymer also has a higher gradient (Young's modulus) than that of the unintercalated polymer, demonstrating that it is not just a transfer of stress that is responsible for the increase but that the intercalated polymer also contributes. Taking the tactoid as a whole, in Figure 3, we see that the stress–strain gradient is significantly greater than that of the unintercalated polymer. Intercalated polymers, which correspond to a large fraction of the polymer molecules, are not present in the non-intercalated “long” PEG system and their absence is the cause of the small Young's modulus increase observed in these nanocomposites.

## 8. Conclusions

We have performed in silico modeling of the interaction of several synthetic polymers with multiple clay platelets, each comprising up to four aluminosilicate sheets. Chemical specificity is preserved thanks to a systematic multiscale approach that commences at the parameter-free density functional level of quantum theory, transferring key data through all-atom classical MD to a coarse-grained dynamical representation that permits the study of the fundamental processes that determine the unusual materials properties of clay–polymer nanocomposites. These processes include the intercalation of molten polymers within montmorillonite tactoids and the subsequent larger-scale assembly of these polymer-entangled tactoids. We have shown that only polymers with highly favorable interactions (hydrogen bonding, for example) can overcome the energy penalty of separating the clay sheets and subsequently intercalating. Our models predict many important properties of these nanocomposites, including realistic X-ray diffractograms and clay layer *d*-spacings, as well as Young's moduli and other materials properties, which are all similar to those reported in experimental work. Furthermore, the processing conditions whereby such materials are synthesized can also be incorporated into these studies; as one example, we showed that the end state from such simulations can depend strongly on the initial conditions, such as whether the clay sheets are already dispersed or integrated into tactoids. As a result, our approach should enable materials scientists and engineers to search systematically for clay–polymer nanocomposites with specified properties, rather than continue to rely on trial and error in laborious experimental studies.

The systematic and chemically specific nature of the modeling and simulation methodology described here makes it immediately applicable to a range of other layered materials, including anionic clays (also known as layered double hydroxides), graphite, and graphene. In addition, our approach can be used to investigate mechanisms of exfoliation by introducing an external shearing force and, with additional parameterizations, to investigate nanocomposites containing surfactants. These developments open the road to accelerated testing and properties assessment of candidate composite materials, provided that progress proceeds in step with rapid experimental advances in creating and characterizing these composite systems. It appears unlikely that algorithmic advances will allow us to resolve the self-assembly of layered nanocomposites on an all-atom level in the near future, primarily due to limitations in clock speed of single computing cores. However, improvements in coarse-grained time integration techniques, together with advances in coupling coarse-grained MD to finite-element models (e.g., as reported by Pfaller et al.[102]) will facilitate a further extension of our approach to yet larger length and time scales, giving more momentum to this growing field of exploration.
