# Supplementary material for: Chemically Specific Multiscale Modeling of Clay–Polymer Nanocomposites Reveals Intercalation Dynamics, Tactoid Self-Assembly and Emergent Materials Properties
Source: Adv Mater. 2014 Dec 9;27(6):966–84. doi: 10.1002/adma.201403361 (PMC4368376; doi:10.1002/adma.201403361)
Supplement: Supplementary file 1 [file adma0027-0966-sd1.pdf]

Copyright WILEY-VCH Verlag GmbH & Co. KGaA, 69469 Weinheim, Germany, 2015.

ADVANCED MATERIALS

Supporting Information

for *Adv. Mater.*, DOI: 10.1002/adma. 201403361

Chemically Specific Multiscale Modeling of Clay–Polymer Nanocomposites Reveals Intercalation Dynamics, Tactoid Self-Assembly and Emergent Materials Properties

*James L. Suter, Derek Groen, and Peter V. Coveney**

Copyright WILEY-VCH Verlag GmbH & Co. KGaA, 69469 Weinheim, Germany, 2014.

Supporting Information

Chemically specific multiscale modeling of clay-polymer nanocomposites reveals intercalation dynamics, tactoid self-assembly and emergent materials properties

James L. Suter Derek Groen
Peter V. Coveney*
Centre for Computational Science, University College London
20 Gordon Street, London, WC1H 0AJ, United Kingdom
E-mail: p.v.coveney@ucl.ac.uk

1 Introduction

In this Supporting Information (SI), we report in detail the methodology we have employed in constructing our multiscale clay-polymer simulation system. Our aim is to retain chemical specificity while extending the time and spatial scales; it is therefore of great importance that the derivation of the interaction parameters is carefully considered. The study described in the main article [1] describes the application of multiscale methods within an investigation of clay-polymer nanocomposites. Here we describe the techniques we have used to generate the coarse-grained potentials, including how we address the sampling problems that can result in less than realistic interaction potentials. We list the simulations we have performed, both at the atomistic (all atom) and coarse-grained level, to create all the parameters required to describe these complex systems. We then show how we validate our models by considering their swelling characteristics. In addition, we provide two animations of 'long' PVA polymer intercalation for which snapshots are shown in Fig. 4 of [1].

2 Coarse-graining overview

We use a systematic ("bottom-up") approach to derive effective pair potentials for a simplified coarse-grained system where groups of atoms are represented by a single interaction site. To do so, we firstly defined a mapping scheme to represent the transformation from a small number of atoms (in this study, less than ten) to a single interaction site. In section 3, we describe the mapping implemented for the polymer and clay systems in this study.

We assume that the total potential energy of the coarse-grained system can be separated into bonded and non-bonded interaction energies; in section 4, we optimise the bonded parameters to match those of the atomistic target system. For the non-bonded interactions (section 5), we have used a combination of techniques to construct the coarse-grained effective pair potentials (which we refer to as interaction potentials), all of which are constructed to reproduce selected properties of fine-grained (atomistic) simulations. In the systems under

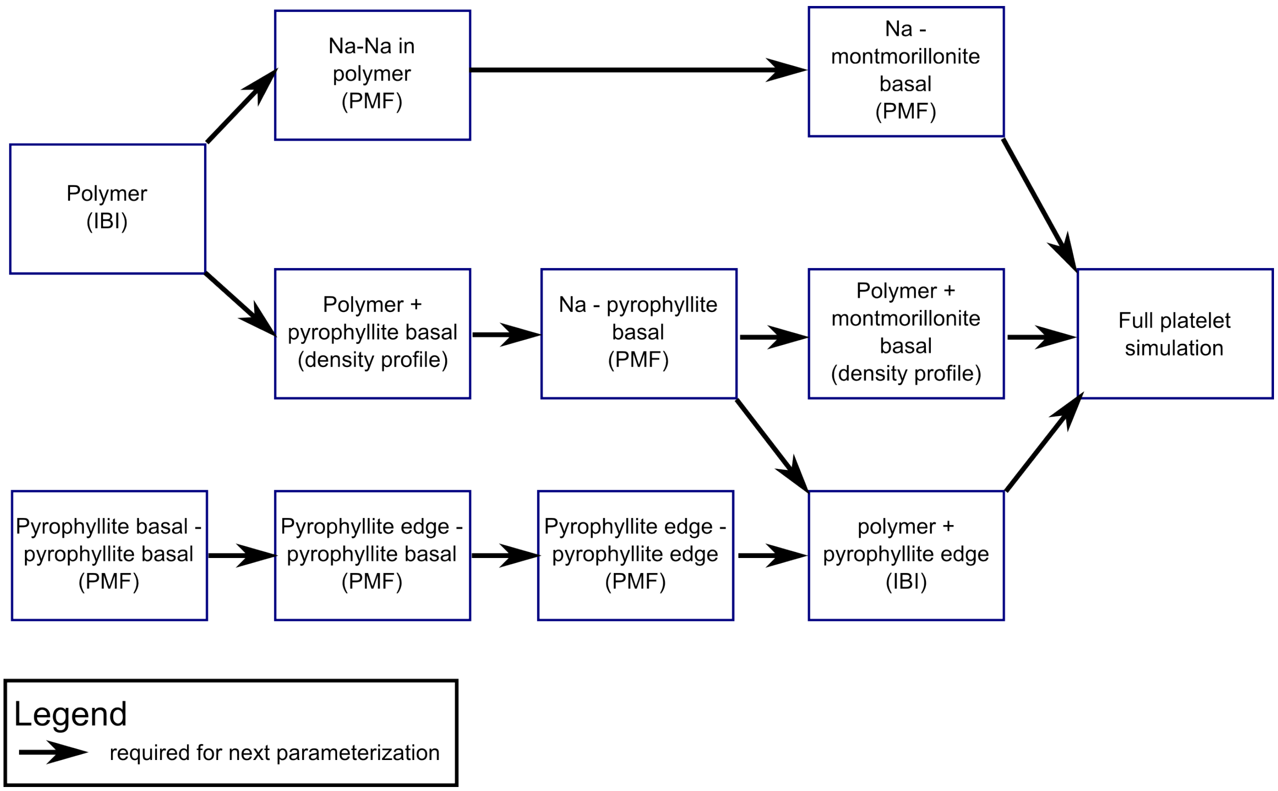

Figure 1: A schematic diagram illustrating the workflow used to compute the large number of interaction potentials required to simulate the coarse-grained montmorillonite platelet-polymer systems described in the main paper [1].

consideration, we need to effectively capture both the properties of the polymer and the changes that occur to the polymer when it is adsorbed on the clay surface.

We have therefore broken down the construction of the interaction potentials into several different stages. Firstly, we have used the Iterative Boltzmann Inversion (IBI) method, described in section 6, to calculate interaction potentials of the pure polymer systems. In this study, we considered two polymers: poly(vinyl) alcohol (PVA) and poly(ethylene) glycol (PEG).

The potentials derived using this method are then subsequently employed in the construction of clay-polymer interaction parameters, by matching the density profiles perpendicular to the clay surface (section 7). Since the interaction of sodium counterions with a clay surface is very strong, we used biased molecular dynamics simulations to construct potentials of mean force (PMF), at both the atomistic and coarse-grained levels, as detailed in section 10, to optimise the interaction potentials. Similarly, the interactions of a clay sheet with other clay sheets cannot be represented by a standard atomistic molecular dynamics simulation; in section 11 we show how we used PMFs to optimise the interactions between clay sheets. A flow chart illustrating the procedure for constructing all the interaction potentials is shown in Figure 1. The simulations, both atomistic (all atom, AA) and coarse-grained (CG), were performed at 500K. All atomistic simulations used the ClayFF [2] and CVFF [3] forcefields to describe the clay and the polymers respectively and were simulated at a pressure of 1 atmosphere (*i.e.* in the same thermodynamic state).

3 Atomistic to coarse-grained mapping

In this section we describe the mapping operator that transforms selected groups of atoms into coarse-grained particles.

3.1 Polymers

In the main article, we perform coarse-grained molecular dynamics of clay interacting with polymer molecules of two different lengths, which we term 'long' and 'short' polymers [1]. The 'long' molecules contain 100 monomer units and the 'short" molecules contain between 31-35 monomer units. The relaxation times of such lengths of polymers are too long to be simulated by atomistic molecular dynamics. Therefore, to derive coarse-grained interaction potentials for polymers from atomistic simulations, we have used relatively short chain polymers of 3 - 4 monomer units, as shown in Figure 2. The short length of these polymer molecules and high temperatures (500K) ensure that we can reach equilibrium within a few nanoseconds of simulation, and can subsequently be used to generate coarse-grained interaction potentials. Through the remainder of the SI, all mention of polymers used for coarse-grained parameterisation refers to these short-chain polymer molecules shown in Figure 2. The longer polymers in the main article ('short' and 'long') [1] use the parameterisations derived from these short-chain polymer molecules.

The mapping of the atomic coordinates into a coarse-grained position for short-chain poly(vinyl alcohol) (PVA) and poly(ethylene) glycol (PEG) is shown in Figure 2. The blue circles enclose the atoms that constitute the terminal polymer coarse-grained particles (polymer$_{\text{terminal}}$) and the green lines enclose the monomer units of the polymer (polymer$_{\text{monomer}}$). The mapping of the enclosed atoms to the coarse-grained position was calculated using the centre-of-mass of these atoms, with an increased weight of the hydroxyl oxygen atom in poly(vinyl alcohol), as we found that the chemically important hydroxyl hydrogen atom was not well represented by a spherical bead at the exact centre-of-mass position. By doubling the weight of the hydroxyl oxygen atom, we found the centre-of-mass position was shifted for PVA by approximately 0.35 Å. Using a spherical cut-off of 6.25 Å (which corresponds to the distance the potential crosses zero in the PVA-clay surface potential), we found that the percentage of the volume of the hydroxyl hydrogen atoms (calculated using its van der Waals radius) covered by the sphere was, on average, approximately 95% when the sphere was located at the shifted centre of mass position, as opposed to 75% on average using the exact centre-of-mass position.

3.2 Clays

In this study, we considered two smectite aluminosilicate clays: the uncharged pyrophyllite $Al_2Si_4O_{10}(OH)_2$ and the charged montmorillonite (where Al^{3+} ions are isomorphically substituted by Mg^{2+} ions, with Na^+ counter ions). For simplicity, we have only considered montmorillonite clays with isomorphic substitution sites in the octahedral layer $Al_{2-x}Mg_xSi_4O_{10}(OH)_2Na_x$.

Each cation (Al^{3+} or Mg^{2+}) in the octahedral layer of the clay is mapped to the centre of a coarse-grained particle. For a single clay sheet, there can be several different coarse-grained particle types depending on where we place the periodic boundary conditions and the residual charge of the clay. For example, if the clay sheet has no sites of isomorphic substitution, its overall charge is zero (*i.e.* a pyrophyllite clay), and if we only consider periodic boundaries on the clay, then there will only be a single coarse-grained particle (which we term clay$_{\text{basal}}$, as it forms the basal (001) surface of the clay). In the case of a clay with an overall negative charge due to substitutions of Al^{3+} by Mg^{2+} (*i.e.* a montmorillonite clay), we define a new coarse-grained particle type, clay$_{\text{charge}}$, which is mapped onto the atomistic structure as the position of the charge substitution (in

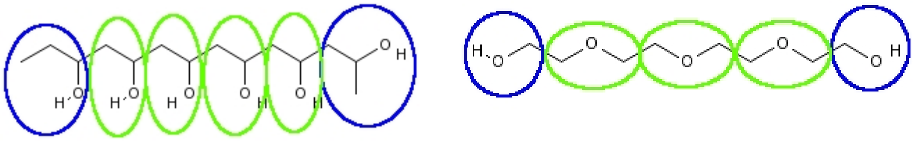

Figure 2: The mapping scheme between the atomistic and coarse-grained representations for the short-chain PVA (left) and PEG (right) molecules used in the parameterisation of the clay-polymer system. The blue circles enclose the terminal units of the polymer ($\text{polymer}_{\text{terminal}}$) while the green ellipses enclose the monomer units of the polymer ($\text{polymer}_{\text{monomer}}$).

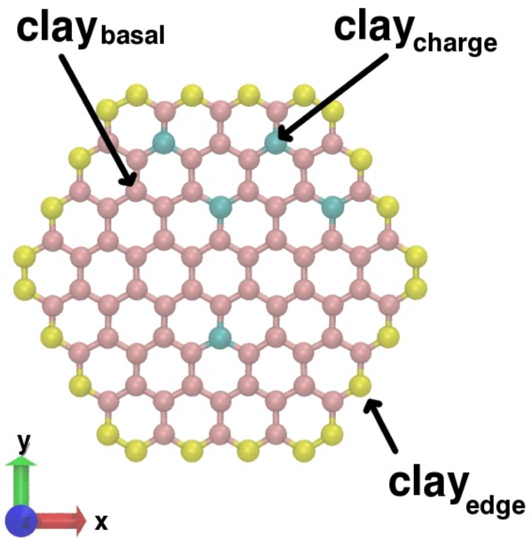

Figure 3: The various coarse-grained clay particle types on a single clay sheet: $\text{clay}_{\text{basal}}$ (pink), $\text{clay}_{\text{charge}}$ (turquoise) and $\text{clay}_{\text{edge}}$ (yellow).

this case, the octahedral Mg^{2+} ions). The charge-balancing ion (Na^{+}) is also mapped to a single clay particle Na_{CG}.

If we place the periodic boundaries away from the clay surface, we need to consider the now exposed edges of the clay (010 and 110). For simplicity, we construct a single coarse-grained particle to represent both these edges, which we term $\text{clay}_{\text{edge}}$, and we do not consider any sites of isomorphic substitution in the clay edges. We have therefore defined 4 different clay coarse-grained particle types: $\text{clay}_{\text{basal}}$, $\text{clay}_{\text{edge}}$, $\text{clay}_{\text{charge}}$ and Na_{CG}. An illustration of the different coarse-grained clay particle types is shown in Figure 3.

4 Coarse-grained bonded degrees of freedom

As stated previously, we use the assumption that we can separate the coarse-grained potential energy into non-bonded and bonded contributions. We firstly calculate the bonded parameters, assuming that the bond, angular and dihedral terms are non-correlated. The initial guesses $U(\zeta)$ are taken from a Boltzmann inverse of the target probability distribution, in a manner akin to Inverse Boltzmann Iteration (see section 5.1):

$$U(\zeta) = -k_B T \ln P_{\mathtt{AA}}(\zeta). \tag{1}$$

where $P_{AA}(\zeta)$ is the Jacobian transformed probability distribution for the atomistic simulation of the required non-bonded degrees of freedom, k_B is the Boltzmann constant, and T is the simulation temperature.

These potentials are then further optimised to increase the agreement in the probability distributions, in the order of bonds, angles and dihedrals, by adding a corrective term to the coarse-grained potentials at each iteration i:

$$U(\zeta, i+1) = U(\zeta, i) + k_B T \ln \left\{ \frac{P_i(\zeta)}{P_{\mathtt{AA}}(\zeta)} \right\}. \tag{2}$$

4.1 Coarse-grained bonded degrees of freedom for clays

The coarse-grained clay particles are connected via harmonic bonds and form a hexagonal network (shown in Figure 3). There are two angles defined for a coarse-grained clay representation: in-plane and out-of-plane. The in-plane angles have an equilibrium value of 120° to maintain the hexagonal ring. The in-plane angles are fitted to the harmonic bond potential: $U = K(\theta - \theta_0)^2$, where K is the angle coefficient and θ_0 is the equilibrium angle. The out-of-plane angles are used to give the clay bending rigidity and to prevent the clay sheet from folding back onto itself. These angles are defined by 2 bonded particles and a third particle on the opposite side of the hexagonal ring, for which the angle, when the clay sheet is flat, is 180 degrees. Deviations from zero indicate the out-of-plane movement of the clay sheet. An illustration of the clay angle definitions is shown in Figure 4. The out-of-plane angle was fitted to a cosine function, $U = K[1 + \cos(\theta)]$, where K is the angle coefficient. As described above, these internal degrees of freedom are firstly calculated from atomistic simulations using direct inversion methods and subsequently iterated until good agreement with atomistic distributions is achieved. The final comparisons between the atomistic and the coarse-grained angles is shown in Figure 5.

Within a single clay sheet, these are the only interactions, *i.e.* there are no non-bonded interactions within the same clay sheet. The out-of-plane angle terms are sufficient to stop the clay sheet from rolling-up or crumpling and interacting with itself. However, we do define non-bonded interactions between different clay sheets (see section 11).

4.2 Coarse-grained bonded degrees of freedom for PVA and PEG polymers

For both the PVA and PEG polymers, the bonded coarse-grained potentials for bonds and angular terms were calculated as tabulated functions, with an interval of 0.1 Å. Spline functions are fitted to these potentials (and all the tabulated potentials described here) within the LAMMPS code. The dihedral potentials for the PEG system were fitted to the following function ("multi/harmonic" entry in Table 1):

$$U(\theta) = \sum_{n=1,5} A_n \cos^{n-1}(\theta) \tag{3}$$

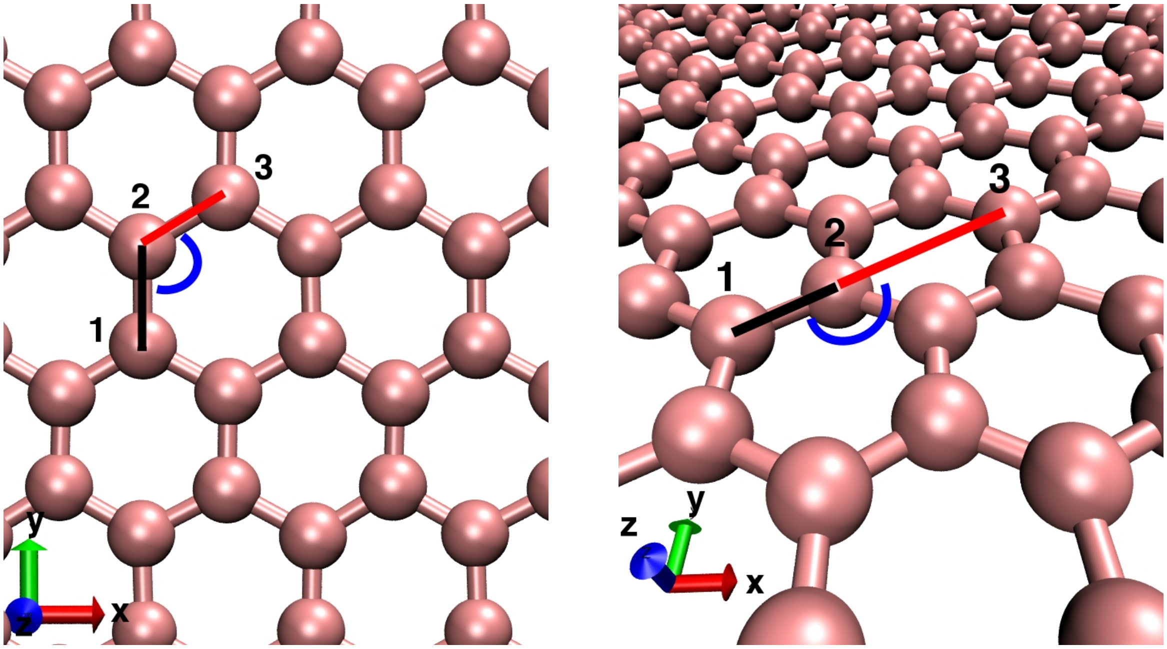

Figure 4: Illustration of the angle types for the coarse-grained clay particles. Left panel: the in-plane angle terms, defined as the angle between bond vector 1-2 (black) and 2-3 (red); the angle is shown in blue, whose equilibrium value is 120 degrees. Right panel: the out-of-plane angle, defined as the angle between bond vector 1-2 (black) and the vector between atoms 2-3 (red). When the clay sheet is flat, the value of this angle (shown in blue) is 180 degrees.

where A_n are five energy coefficients.

For the PVA dihedral potentials, dihedral coefficients were best fitted with the following potential (referred to as "Fourier" in Table 1):

$$U(\theta) = \sum_{i=1,m} K_i[1 + \cos(n_i\theta - d_i)] \tag{4}$$

The agreement in probability distributions for the bonds, angles and dihedral terms of the PVA and PEG polymers are shown in Figures 6 and 7 respectively.

Table 1: A list of non-tabular bonded coarse-grained parameters

parameter type	species involved	description	parameters
in-plane angles	$clay_{basal}$ - $clay_{basal}$ - $clay_{basal}$	harmonic	θ_0=120°, K=8.77 kcal mol^{-1} rad^{-2}
in-plane angles	$clay_{edge}$ - $clay_{basal}$ - $clay_{basal}$	harmonic	θ_0=120°, K=6.7 kcal mol^{-1} rad^{-2}
out-of-plane angles	$clay_{basal}$ - $clay_{basal}$ - $clay_{basal}$	cosine	K=69.58 kcal mol^{-1}
out-of-plane angles	$clay_{edge}$ - $clay_{basal}$ - $clay_{basal}$	cosine	K=50.94 kcal mol^{-1}
PEG dihedral	$polymer_{(ter-mon-mon-mon)}$	multi/harmonic	A_1=0.44, A_2=-0.32, A_3=-0.07,
			A_4=-002, A_5=-0.05 kcal mol^{-1}
PVA dihedral	$polymer_{(ter-mon-mon-mon)}$	Fourier	m=1, K=0.19 kcal mol^{-1}, n_i=2, d=74°
PVA dihedral	$polymer_{(mon-mon-mon-mon)}$	multi/harmonic	A_1 = 0.21, A_2=-0.19, A_3=0.58,
			A_4=-0.46, A_5=-0.09 kcal mol^{-1}

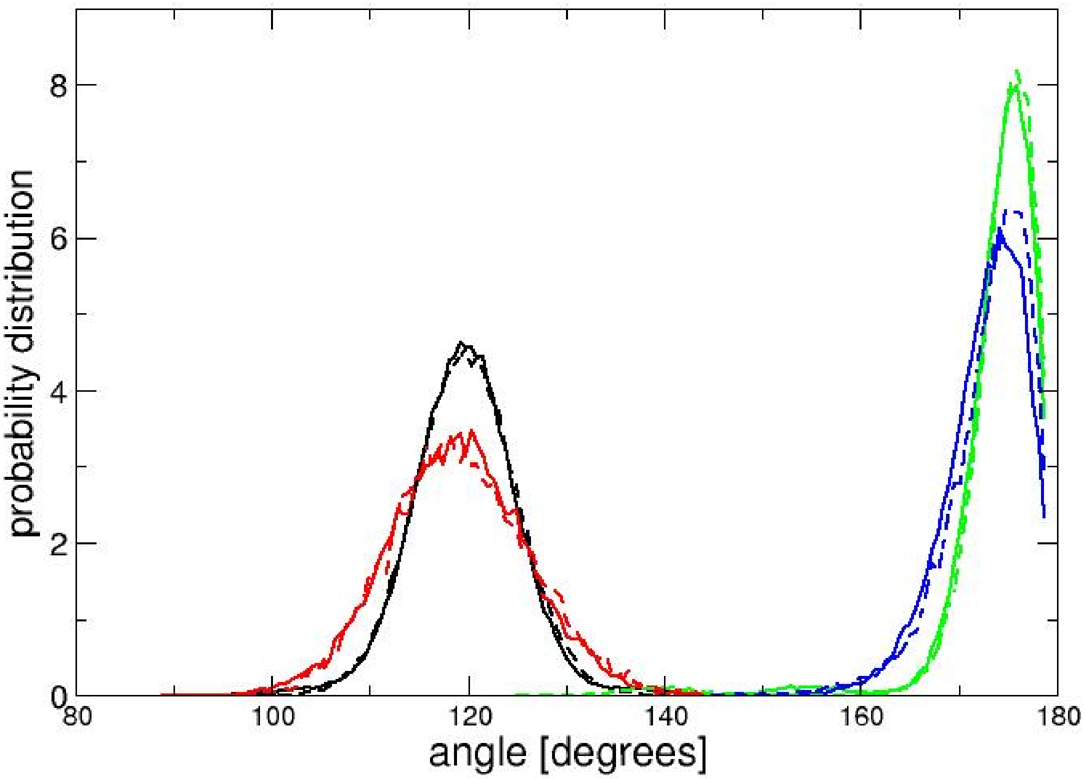

Figure 5: The probability distributions for the in-plane and out-of-plane angles for atomistic and coarse-grained representations of the clay sheet. The solid lines are derived from the atomistic simulations, the dashed lines from the optimised coarse-grained simulations. The black and green lines are for in-plane and out-of-plane angles respectively involving only clay$_{\mathrm{basal}}$ and clay$_{\mathrm{charge}}$ particles while the red and blue lines are for in-plane and out-of-plane angles respectively involving clay$_{\mathrm{edge}}$ particles.

5 Non-bonded interaction potentials

In Figure 1, we show the work-flow involved in computing all the interaction potentials required for the clay platelet simulations described in the manuscript. In the work-flow, within each box we have indicated the potentials generated (and subsequently used to produce the next set of potentials in the work-flow) and the method employed. For the interaction potentials between polymer molecules (first box), we have used the Iterative Boltzmann Inversion (IBI) method 5.1. Subsequent interaction potentials were generated through matching density profiles perpendicular to the clay surface and to potentials of mean force calculated from constrained molecular dynamics simulations. All non-bonded interactions were of tabular form, with an interval of 0.1 Å for potentials generated using IBI and an interval of 0.25 Å for all other potentials.

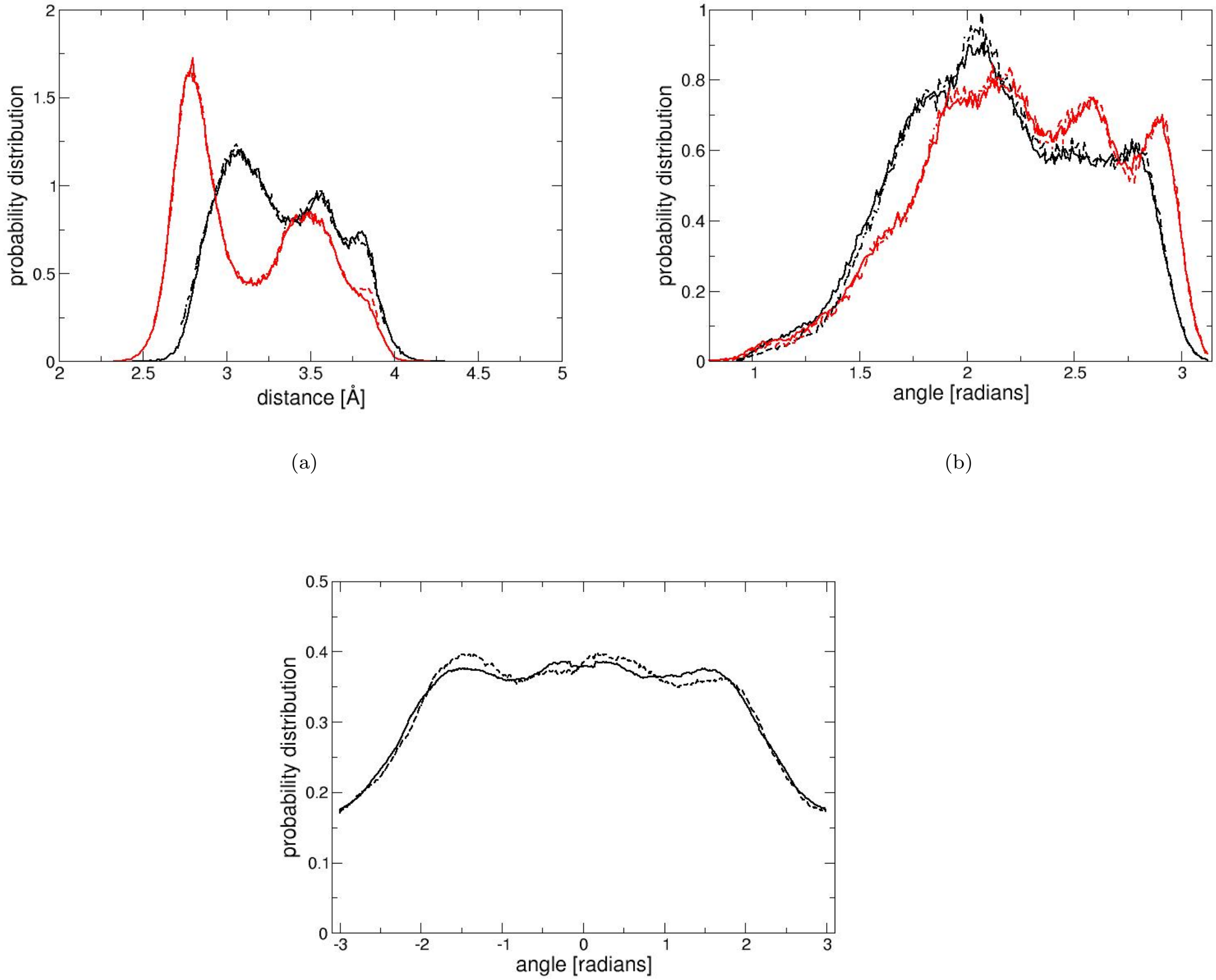

(a) (b)

(c)

Figure 6: The bonded degrees of freedom for the PVA polymer at the atomistic (solid lines) and coarse-grained level (dashed lines). (a) the bonds in the system, the black lines are polymer$_{\text{terminal}}$-polymer$_{\text{monomer}}$ bonds and the red lines are polymer$_{\text{monomer}}$-polymer$_{\text{monomer}}$. (b) the angles in the system, the black lines are polymer$_{\text{terminal}}$-polymer$_{\text{monomer}}$-polymer$_{\text{monomer}}$ angles and the red lines are polymer$_{\text{monomer}}$-polymer$_{\text{monomer}}$-polymer$_{\text{monomer}}$ angles. (c) the dihedrals in the polymer$_{\text{monomer}}$-polymer$_{\text{monomer}}$-polymer$_{\text{monomer}}$-polymer$_{\text{monomer}}$ system. In this, and other figures illustrating the matching of coarse-grained and atomistic distributions, the agreement between the two levels is such that the curves effectively lie on top of each other and cannot be discerned.

5.1 Iterative Boltzmann Inversion

IBI is a structure-based coarse graining technique. We take an initial potential derived from atomistic simulations, and apply it to a coarse-grained system [4]. We then run a coarse-grained (CG) simulation and extract the resulting radial distribution functions (RDFs) from each type of interaction. We compare coarse-grained

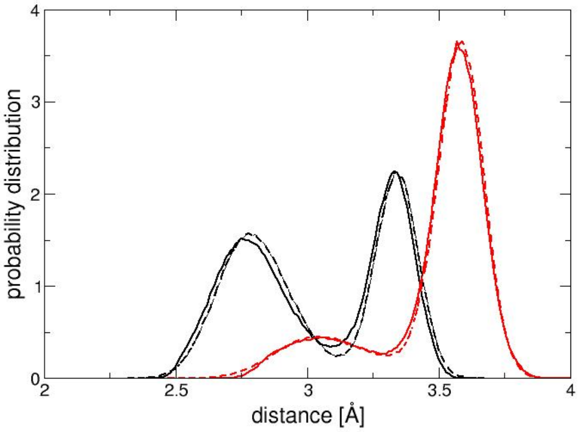

(a)

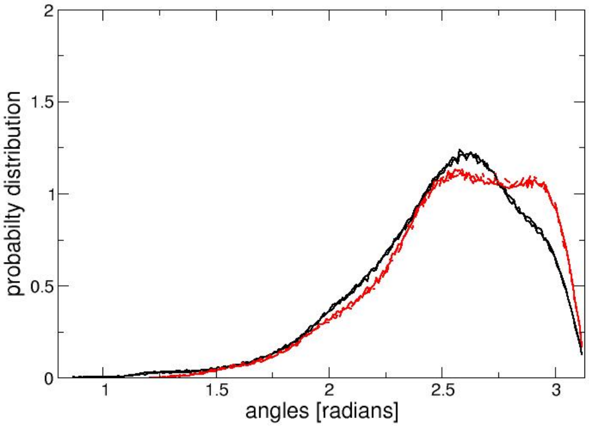

(b)

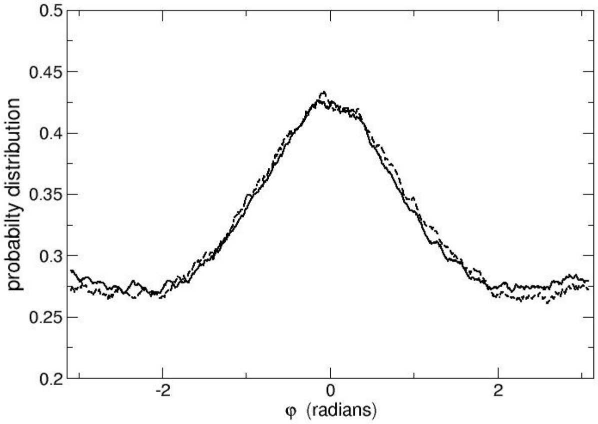

(c)

Figure 7: The bonded degrees of freedom for the PEG polymer at the atomistic (solid lines) and coarse-grained level (dashed lines). (a) the bonds in the system, the black lines are polymer$_{\text{terminal}}$-polymer$_{\text{monomer}}$ bonds and the red lines are polymer$_{\text{monomer}}$-polymer$_{\text{monomer}}$. (b) the angles in the system, the black lines are polymer$_{\text{terminal}}$-polymer$_{\text{monomer}}$-polymer$_{\text{monomer}}$ angles and the red lines are polymer$_{\text{monomer}}$-polymer$_{\text{monomer}}$-polymer$_{\text{monomer}}$ angles. (c) the dihedrals in the system (there is only one type).

RDFs against target RDFs obtained from atomistic runs, and then update the coarse-grained potential U for each distance r according to the following rule:

$$U(r, i+1) = U(r, i) + k_B T \ln \left\{ \frac{g(r, i)}{g_{\mathtt{AA}}(r)} \right\}. \tag{5}$$

Here $U(r, i+1)$ is the new coarse-grained potential, $U(r, i)$ the potential used previously, k_B the Boltzmann constant, T the temperature, $g(r, i)$ the RDF obtained from the coarse-grained run and $g_{\mathtt{AA}}(r)$ the atomistic target RDF. Our IBI implementation does not use any *ad hoc* acceleration coefficient, as described for example in [5]. Once the potential is updated, we run a new coarse-grained simulation, repeating this process until the

RDFs have converged.

To ensure rapid propagation of the method, we run each coarse-grained simulation long enough to obtain a reasonable statistical validility, but short enough to ensure rapid advancement of the algorithm. For the systems we study here, we chose to run the coarse-grained simulations for a minimum of 250,000 time steps. The potentials were smoothed using a Hanning window of length 13 values.

5.2 Pressure correction

In many cases, IBI results in a coarse-grained system that reproduces the atomistic target RDF, but which has an internal pressure that differs greatly from the reference system. To compensate for this, we apply a pressure correction term P_c in conjunction that each IBI iteration, adjusting the iterative rule such that:

$$U(r, i+1) = U(r, i) + k_B T \ln \left\{ \frac{g(r, i)}{g_{\mathtt{AA}}(r)} \right\} + P_c. \tag{6}$$

Here, for each interaction between every particle type a and b we apply a pressure correction as described, for example, by Fu *et al.* [5]:

$$- \left[\frac{2\pi\rho^2}{3r_{\text{cut}}} \int_0^{r_{\text{cut}}} r^3 g^k(r) dr \right] A^k \approx (P - P_{\text{target}}) \, . \tag{7}$$

In this step we calculate the pressure correction factor A^k, using various calculated properties including the density (ρ), the cutoff radius (r_{cut}), the positional values (r), the RDF ($g^k(r)$), and the current and target pressure of the system (P and P_{target} respectively). We multiply the pressure correction factor with a scaling factor (typically between 0 and 1) to optimally control the effect of the pressure correction per IBI iteration.

6 Derivation of polymer interaction parameters

Using the IBI scheme defined above, we derived coarse-grained interaction potentials for the polymer systems. In the subsections below, we describe the atomistic target system and show the agreements between the atomistic and the coarse-grained systems.

6.1 Poly(vinyl) alcohol

Atomistic details: 4400 atoms (88 molecules of 50 atoms, each molecule corresponding to 2 terminal units and 4 monomer units). The total simulation time was 5ns, corresponding to 5,0800,000 timesteps with a timestep of 1fs. The system was simulated in an NpT ensemble, with a pressure of 1.0 atmosphere and a temperature of 500K maintained by a Nosé-Hoover thermo/barostat. The average simulation supercell lattice parameters for the x, y and z directions for the atomistic system was 37.23 Å. The simulation had an average pressure of -26.8259 ± 1043.18 atmospheres.

The corresponding coarse-grained system contained 588 atoms (50 molecules of 6 coarse-grained units) and was run under NVT conditions with the x, y and z lattice parameters fixed at 37.23 Å. The matching radial distribution functions for the coarse- and fine-grained systems are shown in Fig. 8. The average pressure in the coarse-grained system was -41.3 ± 172 atmospheres. Each iteration of the coarse-grained simulation was for 250,000 timesteps with a timestep of 5fs. The matching radial distribution functions for the coarse- and fine-grained systems are shown in Fig. 8.

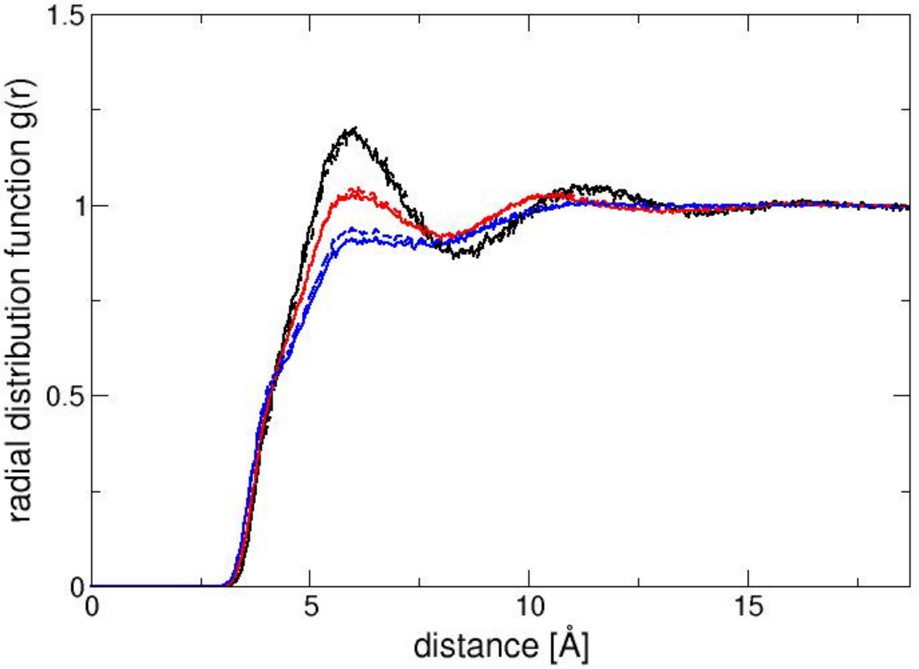

Figure 8: The radial distribution functions at the atomistic (solid lines) and coarse-grained levels (dashed lines) for PVA. Black lines are the g(r) between polymer$_{\text{terminal}}$ and polymer$_{\text{terminal}}$, red lines are the $g(r)$ between polymer$_{\text{terminal}}$ and polymer$_{\text{monomer}}$and blue lines are the $g(r)$ between polymer$_{\text{monomer}}$ and polymer$_{\text{monomer}}$.

6.2 Poly(ethylene) glycol

Atomistic details: 33728 atoms (1088 molecules of 31 atoms, each molecule corresponding to 2 terminal units and 3 monomer units). The system was kept at a constant pressure of 1.0 atmosphere and a temperature of 500K via a Nosé-Hoover thermo/barostat. The total simulation time was 4.94ns, corresponding to 4.943,000 timesteps with a timestep 1.0fs. The average x, y, z simulation supercell lattice parameters was 76.7166 Å, with an average pressure of 1.38 ± 249.315 atmospheres.

The matching radial distribution functions for the coarse and fine grained systems are shown in Fig. 9. Each iteration of the coarse-grained simulation was for 250,000 timestep with a timestep of 5fs. The average pressure in the coarse-grained system was -8.168 ± 106.47 atmospheres.

7 Polymer-surface interactions

At the coarse-grained level, the interaction energy between the clay sheet and polymer is represented by an interaction potential; as we require flexible (and indeed mobile) clay sheets we cannot use a simple z dependent potential (with the clay sheets as walls fixed on the xy plane). We found that the IBI method is, however, unstable when used to create pair-wise interaction potentials in the vicinity of surfaces. In this case, the isotropic environment normally encountered in IBI method is not found. Instead a molecule adsorbing on the surface interacts with a large number of clay surface atoms of varying distance. The extension of systematic

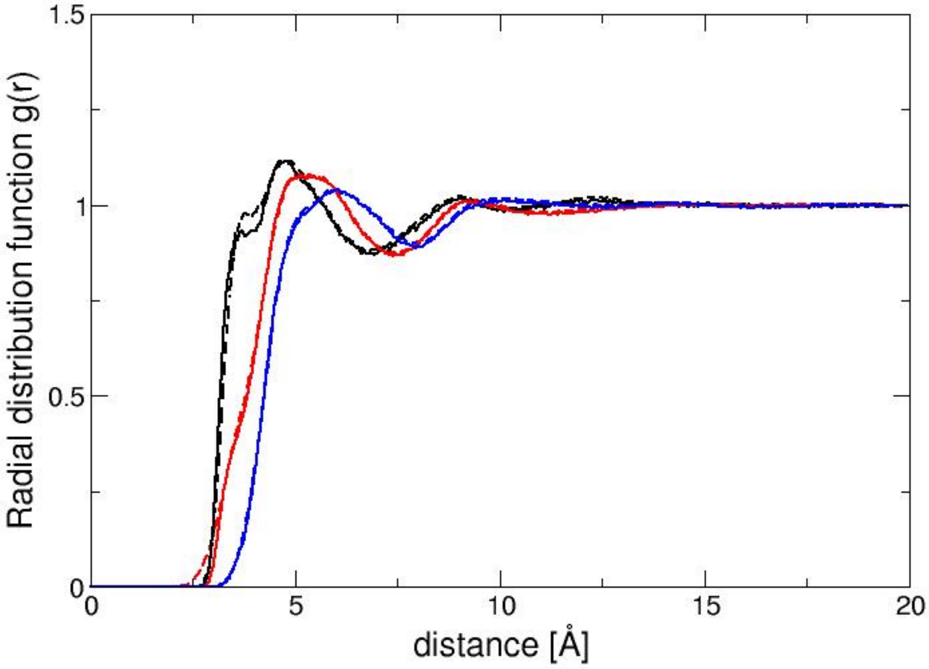

Figure 9: The radial distribution functions g(r) at the atomistic (solid lines) and coarse-grained levels (dashed lines) for PEG. Black lines are the g(r) between polymer$_{\mathrm{terminal}}$ and polymer$_{\mathrm{terminal}}$, red lines are between polymer$_{\mathrm{terminal}}$ and polymer$_{\mathrm{monomer}}$and blue lines are between polymer$_{\mathrm{monomer}}$ and polymer$_{\mathrm{monomer}}$.

coarse-grained models to polymersolid interfacial systems is a challenging research area precisely because of these extra complexities due to the presence of interfaces [6].

The conformations of adsorbed polymer chains are best described by the density profile perpendicular to the surface. This captures the adsorption properties of the polymer, such as the layer thickness and density. The radial distribution functions do not capture these features. We therefore derive coarse-grained polymer-clay interaction parameters which reproduce the atomistic density profile perpendicular to the clay surface. Our target atomistic system is a clay sheet lying in the xy plane, with periodic boundaries located on the clay sheet, and polymer molecules above and below the sheet. A snapshot illustrating the atomistic simulation setup is shown in Figure 10. Details on the atomistic systems are given below.

To generate the coarse-grained interaction potentials we cannot replace $g(r)$ with $\rho(z)$ in equation 6. Instead, we need to modify the energy correction at each step in the IBI equation (eqn. 6) to reflect the complex multiple interactions with the surface atoms of an adsorbing molecule. For a coarse-grained polymer particle, the particle-clay surface distances will be a function of the distance perpendicular to the surface z.

We firstly compute the difference between the atomistic density profile (ρ_{AA}) and the coarse grained density profile (ρ_{CG}) (note that we normalise the density profiles to be 1 at large separations, analogous to $g(r)$). We define $S(r)$ as the number of particles of distance r from a clay sheet atom (counted within an α-interval). $S(r)$ is calculated for every slice of thickness α in the z direction: $S(z, r)$. In total there are N intervals in both z and r. We wish to know the shortest distance of the polymer particle to the clay sheet at each α interval in the z direction; we achieve this by creating a new function $S1(z, r)$, which is a transformation of $S(z, r)$ to

only include values when the integral of $S(z, r)$ with respect to r is less than or equal to one.

We defining a function $F(z)$ that provides a one dimensional mapping between the distance in the z direction and the first atom / particle distance,

$$F(z_k) = \sum_{l=0}^{N} \frac{S1(z_k, r_l)}{\sum_{m=0}^{N} S1(z_m, r_l)}. \tag{8}$$

We then take into account the additional long range interactions. To calculate the update $V(r_k)$ in the IBI equation, we loop through N values, starting with the highest z distances and computing the update according to the pair of equations:

$$V(r_k) = k_B T \ln\left(\frac{\rho_{\mathrm{CG}}(z_k)}{\rho_{\mathtt{AA}}(z_k)}\right) \times F(z_k) - \sum_{j=k+1}^{N} \sum_{l=0}^{N} S(z_l, r_j) \times V(r_j) \tag{9}$$

$$U(r_k, i+1) = U(r_k, i) + \gamma V(r_k) \tag{10}$$

The difference in density profile is transformed into an r dependency by the function $F(z)$. Equation 9 requires a correction from already computed $V(r_k)$ values, i.e. at longer distances than the $V(r_k)$ value being computed, which are scaled by the distance matrix $S(z, r)$ and subtracted from the current computed value. In this way, we take into account the additional interactions with the surface clay atoms / particles that result as the polymer approaches the surface. The final update is then added to the polymer-clay interaction potential from the previous iteration, with a scaling factor γ to increase the stability of the iterative process. In our simulations, γ varied between 0.1 to 0.5. This method was used to generate the density $\rho(z)$ that agrees with the atomistic target within approximately 20 iterations, within a reasonable error.

8 Polymer with pyrophyllite basal surface

To compute the interaction potential between the coarse-grained polymer and clay$_{\mathrm{basal}}$ particles, we simulated a reference atomistic model of a periodic pyrophyllite clay with polymer above and below the clay sheet. A snapshot from this simulation is shown in figure 10.

8.1 PVA with pyrophyllite basal surface

Atomistic details: 9630 atoms. The system contained 167 PVA molecules and was simulated in the NpT ensemble (1 atmosphere and 500K). The average lattice parameters were 41.7 × 36.0 × 75.9 Å^3. The temperature was maintained at 500K and 1 atmosphere, and was simulated for 19,600,000 timesteps at 1.0fs (19.6ns). The coarse-grained system was run in a NVT ensemble, with lattice parameters corresponding to the average lattice parameters for the atomistic system. The coarse-grained system contained 1130 coarse-grained particles. Each coarse-grained iteration was run for 12.5 ns with a timestep of 5fs. The matching density profiles perpendicular to the surface are shown in Figure 11.

8.2 PEG with pyrophyllite basal surface

Atomistic details: 6116 atoms. The average lattice parameters were 41.7 × 36.0 × 52.97 Å^3. The temperature was maintained at 500K and 1 atmosphere, and was simulated for 2,640,000 timesteps at 1.0fs (2.64ns). The coarse-grained system was run in a NVT ensemble, with lattice parameters corresponding the average lattice parameters for the atomistic system. There were 696 coarse-grained particles. Each iteration was run for 12.5 ns with a timestep of 5fs. The matching density profiles perpendicular to the surface are shown in Figure 11.

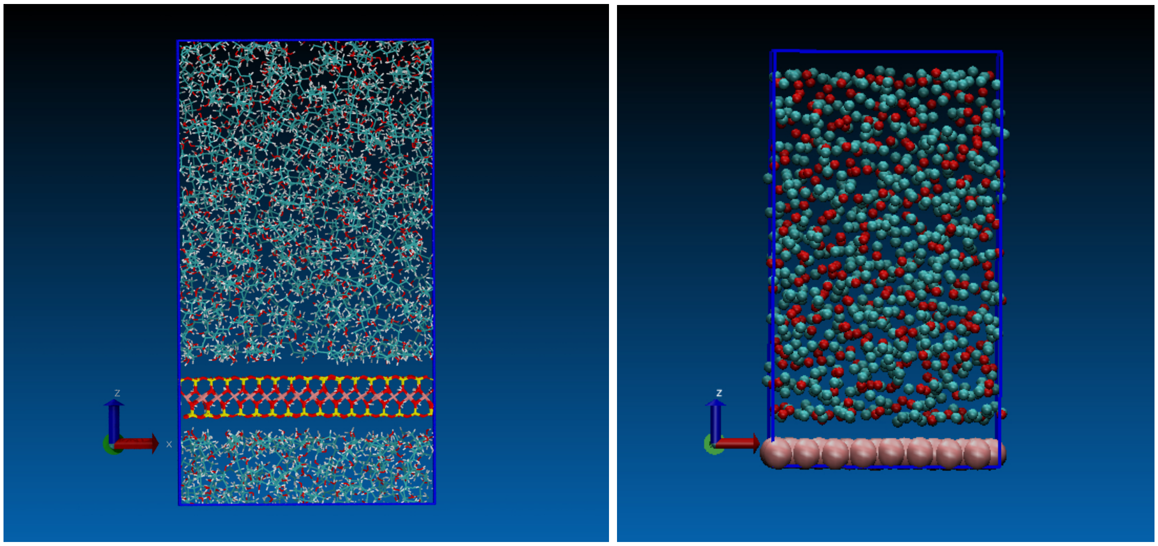

Figure 10: The atomistic reference system and its coarse-grained equivalent for parameterising the polymer-$clay_{basal}$ interaction potentials. The left hand panel is an atomistic simulation of a pyrophyllite clay with PVA molecules. The colours are as follows: red = oxygen, white = hydrogen, green = carbon, yellow = silicon and pink = aluminium. The right hand panel is the coarse-grained equivalent, for which the polymer-$clay_{basal}$ potentials were altered until the density profiles perpendicular to the surface matched. The colours are as follows: pink = $clay_{basal}$, red = $polymer_{terminal}$ and green = $polymer_{monomer}$. The blue lines are periodic boundaries.

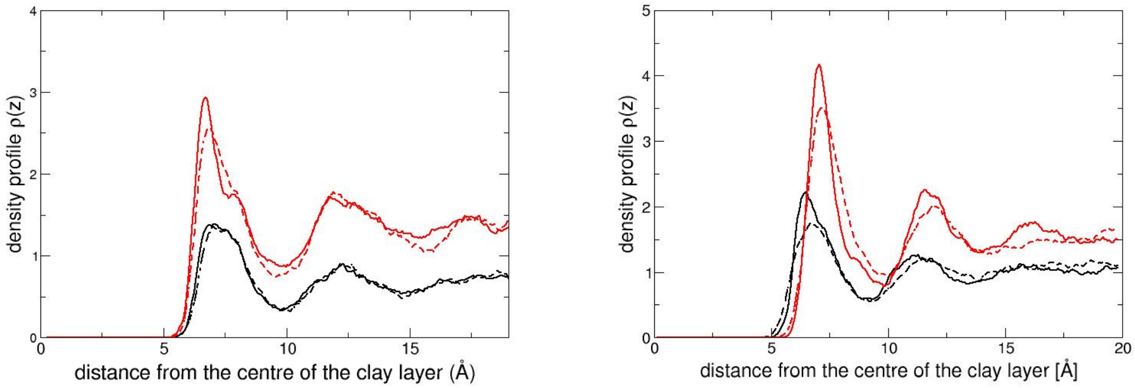

Figure 11: Density profiles perpendicular to the pyrophyllite surface for PVA molecules (left panel) and PEG molecules (right panel). The density profile for $polymer_{monomer}$ are the red lines (solid for atomistic, dashed for coarse-grained systems) and the density profile for $polymer_{terminal}$ are the black solid lines for atomistic and black dashed lines for coarse-grained systems. The density profiles were normalised to one during the iteration process, but are separated here to aid visualisation.

9 PEG/PVA polymer with pyrophyllite clay edges

To simulate the interaction potentials of the coarse-grained clay edges with the polymers, we simulated an atomistic system of a small single pyrophyllite clay sheet immersed in a polymer melt. The platelet had a

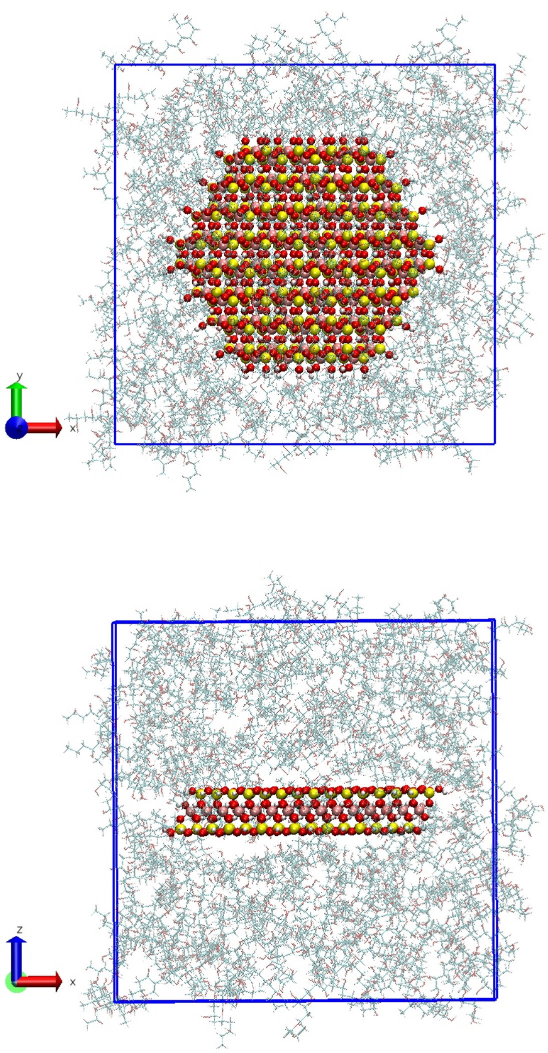

Figure 12: The initial setup of the atomistic simulation to compute the interaction potentials between the polymer and the clay edges. The polymer atoms are displayed as translucent to aid visualisation of the clay sheet. The periodic boundaries are shown in blue; the dimensions are 60 × 60 × 60 Å^3. The Left panel is the visualisation of the xy plane and the right panel is of the xz plane. The colours of the atoms are the same as in Figure 10.

diameter of approximately 40 Å. It was placed in a box of initial dimensions 60 × 60 × 60 Å^3, lying on the xy plane. The clay consisted of 1280 atoms. Short-chain polymers (shown in Figure 2) were inserted into the simulation box using the Monte Carlo growth method described in the main article [1], up to a density of approximately 0.6 g ml^{-1}. In total, 252 PVA molecules (each consisting of 50 atoms) and 327 PEG molecules (each consisting of 31 atoms) were inserted into their respective systems. A snapshot of the starting structure for the PVA-clay system is shown in Figure 12. After energy minimisation, the atomistic simulations were performed for 2ns at 500K and 1 atmosphere. The final lattice dimensions are for clay-PVA: 54.6 × 54.6 × 54.6 Å^3 and for clay-PEG: 52.1 × 52.1 × 52.1 Å^3.

We used IBI to optimise the polymer-clay$_{\mathrm{edge}}$ interaction potentials to match the radial distribution functions between the atomistic and the coarse-grained levels (see Figure 13). Each coarse-grained iteration was for

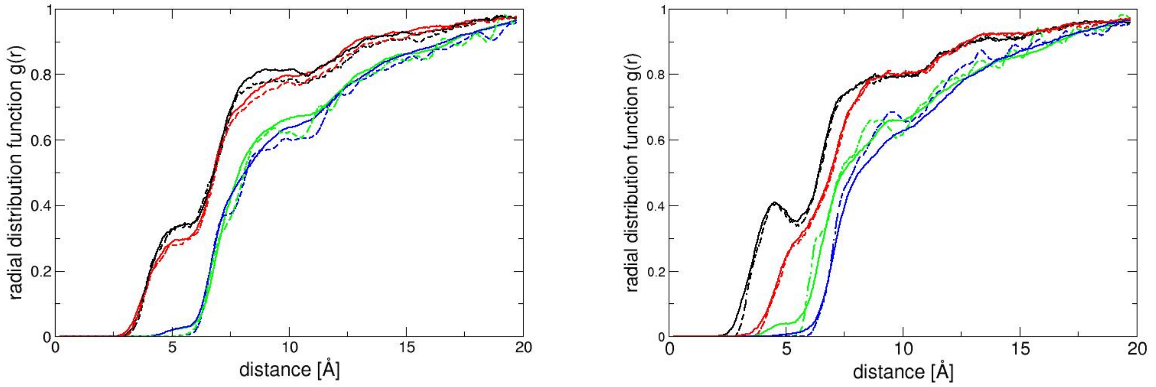

Figure 13: The radial distribution functions for the atomistic level (solid lines) and the coarse-grained level (dashed lines) for PVA (left panel) and PEG (right panel). The $clay_{edge}$-$polymer_{terminal}$ g(r) are the black lines, the $clay_{edge}$-$polymer_{momomer}$ are the red lines. We also show the g(r) for the $clay_{basal}$ coarse-grained types with $polymer_{terminal}$ (green) and $clay_{basal}$ coarse-grained types with $polymer_{monomer}$ (blue), which were parameterised previously by fitting to the density profile perpendicular to the clay surface (section 7).

250,000 timesteps at 5 fs. We also display the radial distribution functions between the coarse-grained $clay_{basal}$ and the polymer particles, which were parameterised previously by fitting to the density profile perpendicular to the clay surface (section 7) and hence have not been fitted to the radial distribution functions; we see in Figure 13 that the agreement between the atomistic and coarse-grained levels for these distributions is good.

10 Na potentials

In our previous atomistic simulations of synthetic polymers interacting with pyrophyllite and montmorillonite surfaces [7], we have found that sodium ions are normally adsorbed on the clay surface for the duration of the simulation. We therefore needed to use a biased sampling technique such that we can fully determine the clay-Na_{CG} interaction potentials. Once we determined the interaction potentials of sodium ion with coarse-grained polymer, we calculated the PMF of a sodium ion adsorbing onto a pyrophyllite surface, and then onto a charged montmorillonite surface.

10.1 Polymer-Na_{CG} potentials

We calculated the interaction potentials between PVA and PEG polymer species and sodium ions by using the IBI technique to match the g(r) between the polymer and sodium ion. To construct the atomistic system, we inserted a sodium and a chloride ion into the equilibrated bulk polymer system described in section 6. We found that when immersed in PVA, the sodium and chloride ions were fully solvated by the PVA polymers; however, the sodium and chloride ions form ion pairs with the PEG polymer (which has a lower dielectric constant than PVA). This effect was replicated in the coarse-grained system. The radial distribution functions at the atomistic and coarse-grained levelsare shown in Figure 14.

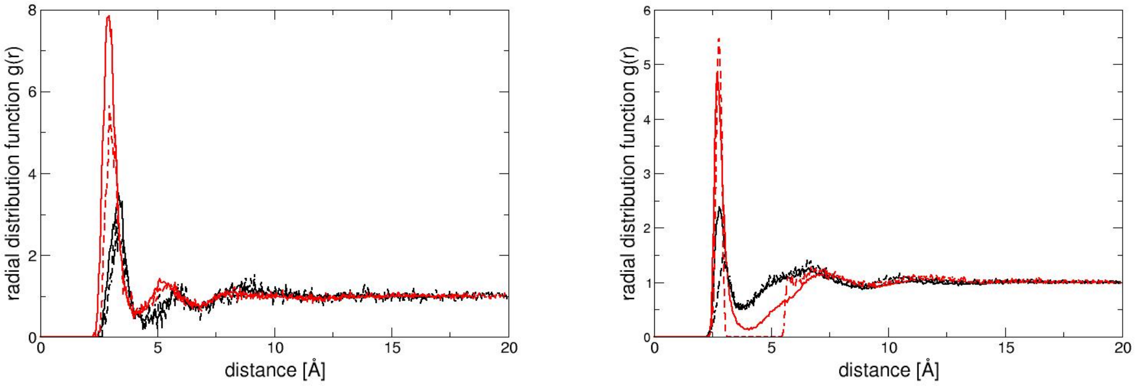

Figure 14: The radial distribution functions for Na and polymer atoms at both the atomistic (solid lines) and the coarse-grained level (dashed lines) for the PVA polymer (left panel) and PEG polymer (right panel). The black lines are the g(r) for polymer$_{\mathrm{terminal}}$-Na$_{\mathrm{CG}}$ and the red lines are the g(r) for polymer$_{\mathrm{monomer}}$-Na$_{\mathrm{CG}}$

10.2 Clay$_{\mathrm{basal}}$-Na$_{\mathrm{CG}}$ potentials

To calculate the clay$_{\mathrm{basal}}$-Na$_{\mathrm{CG}}$ interaction potential, we matched the atomistic and coarse-grained PMF for a sodium ion adsorbing on an uncharged (pyrophyllite) clay surface. In the atomic system, a charge balancing chloride ion is also present. This chloride ion was initially placed at least 30 Å from the sodium ion and the forces it experienced during the simulation were not integrated (leading to the ion remaining stationary, away from the clay sheets). In this case, the reaction coordinate for the PMF calculation is the difference in the z coordinates from the clay sheet (calculated as the average z coordinate of the octahedral aluminium atoms) to the sodium ion. An illustration of this process is shown in Figure 15. The PMF calculation is achieved using umbrella sampling, which requires an ensemble of simulations, each constrained to a value on the reaction coordinate using a harmonic potential. The PMF can then be reconstructed using the weighted histogram analysis method (WHAM) [8].

Initially, the sodium ion was inserted into the final snapshot of the atomistic simulation of the polymer adsorbed onto the pyrophyllite surface (section 9), with the chloride ion inserted as described as above. A harmonic constraint of 4.7 kcal mol^{-1} Å^{-2} was used to restrain the sodium ion at each constraint value. As the restraint only acted on the difference in z coordinates, the sodium ion was free to move in the xy plane. The sodium ion was manually moved to the exact constraint value at the beginning of each ensemble run. The interval between constraint values was 0.25 Å, ranging from 20 Å to 2.5 Å (70 constraint values). The lowest constraint values are within the van der Waals radius of the clay surface atoms. Each constraint run lasted for 100,000 timesteps with a timestep of 1.0fs after initial energy minimisation. The coarse-grained ensemble runs were performed for 2,500,000 timesteps at a 5fs timestep. The update to the clay$_{\mathrm{basal}}$-Na$_{\mathrm{CG}}$ potential was computed by equation 10. The atomistic and coarse-grained PMFs for a sodium ion adsorbing on a pyrophyllite surface in PEG and PVA polymer are shown in Figure 16.

10.3 Clay$_{\mathrm{charge}}$-Na$_{\mathrm{CG}}$ potentials

To calculate the interaction parameters for the sodium ion interacting with a charge site in the clay sheet, we computed the PMF of a sodium ion adsorbing directly onto a charge site in the clay framework (a Mg^{2+} ion

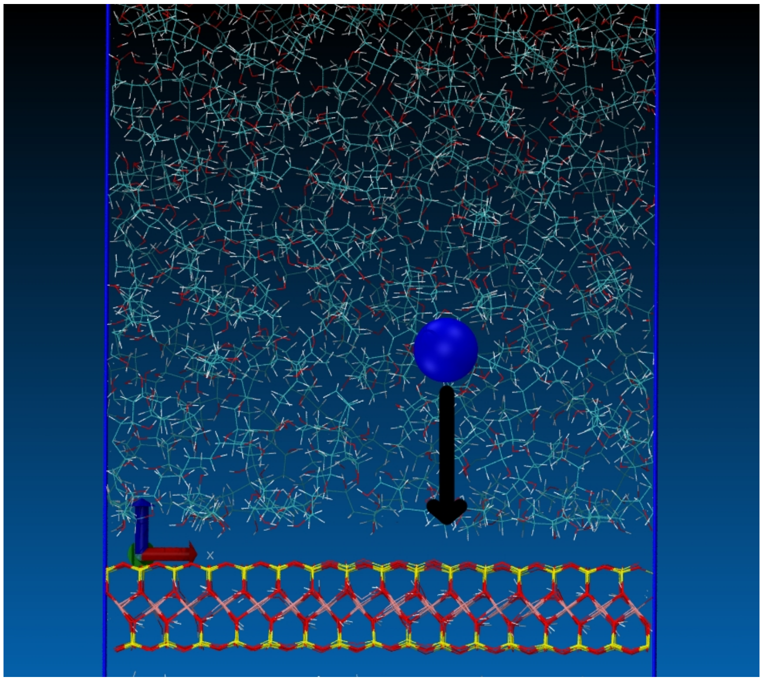

Figure 15: An illustration of the potential of mean force calculation of a sodium ion adsorbing onto a pyrophyllite clay sheet, used to compute the $clay_{basal}$-Na_{CG} interaction potential. The black arrow is the reaction coordinate for the PMF, which corresponds to the difference in z coordinate between the centre of mass of the clay sheet and the sodium ion.

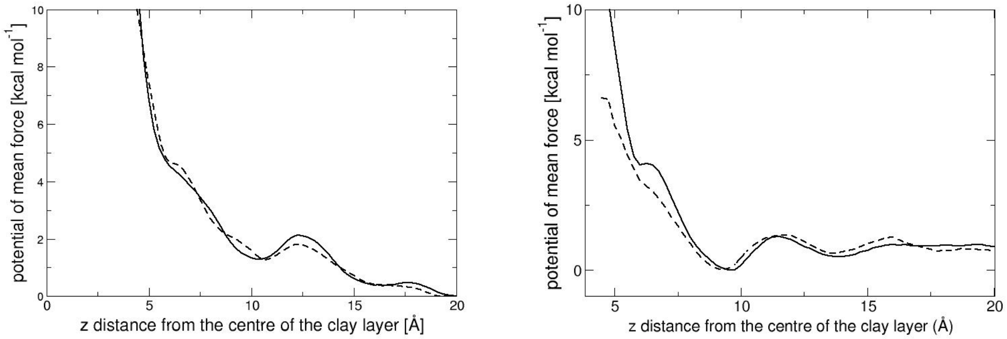

Figure 16: The potential of mean force for adsorption of a sodium ion onto a pyrophyllite clay surface at the atomistic level (solid lines) and the coarse-grained level (dashed lines) for PVA (left panel) and PEG (right panel).

isomorphic substitution in the octahedral layer, *i.e.* a montmorillonite clay). The reaction coordinate is the distance from the charge site (i.e Mg^{2+} ion / coarse-grained $clay_{charge}$ ion) and the sodium ion. The interval between constraint values was 0.25 Å, ranging from 20 Å to 2.5 Å (70 constraint values) with a harmonic constraint of 4.7 kcal mol^{-1} Å^{-2} used to constrain the position of sodium ion. IBI was used to update the $clay_{charge}$-Na potential at each update. The atomistic and coarse-grained PMFs for sodium adsorbing on a montmorillonite surface in PEG and PVA polymer are shown in Figure 17.

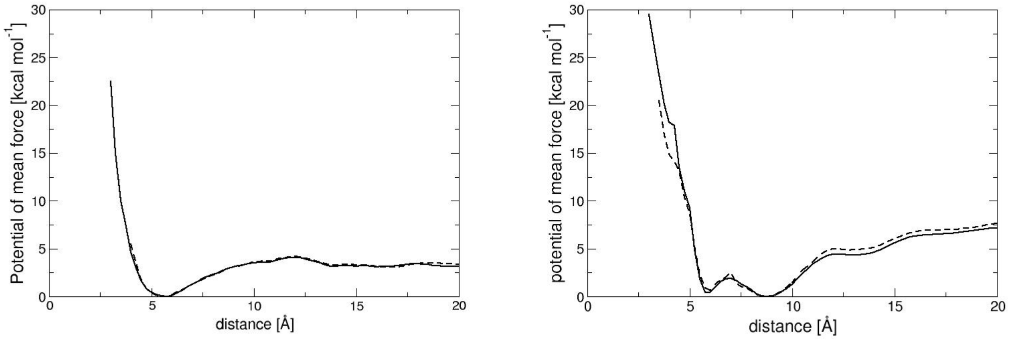

Figure 17: The potential of mean force for adsorption of a sodium ion onto a montmorillonite clay surface at the atomistic level (solid lines) and the coarse-grained level (dashed lines) for PVA (left panel) and PEG (right panel).

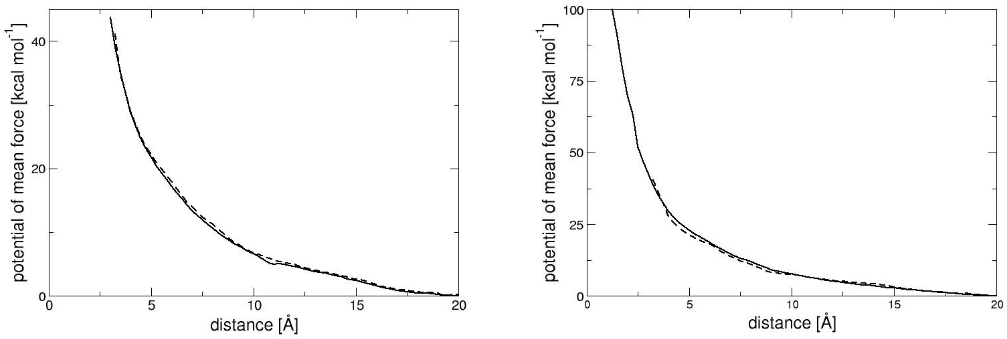

Figure 18: The potential of mean force as a function of the distance between two sodium ions in PVA (left) and PEG (right). The atomistic PMF are solid lines and the coarse-grained PMF are dashed lines.

10.4 Na_{CG}-Na_{CG} potentials

To calculate the interaction between two sodium ions in the polymer, we have performed a PMF calculation where the reaction coordinate is the distance between two sodium ions. We manually inserted two sodium ions and two chloride ions into the final snapshot of the neat polymer simulation. The chloride ions were placed over 25 Å away from the sodium ions and their forces were not integrated during the molecular dynamics simulation to render them stationary. One of the sodium ions was tethered using a strong harmonic potential of 50 kcal mol^{-1} Å^{-2} to ensure the sodium ions did not drift towards the chloride ions. We performed an ensemble of 70 simulations, each with a harmonic potential of 4.7 kcal mol^{-1} Å^{-2} constraining the distance between the sodium ions to a value ranging from 20 Å to 2.5 Å. Each ensemble simulation lasted 0.5ns. The resulting potential of mean force at the atomistic and coarse-grained levels, with optimised Na_{CG}-Na_{CG} interaction potential, is shown in Figure 18.

11 Sheet-sheet potentials

To generate interactions between clay sheets, we need to overcome the sampling problems encountered with a clay sheet interacting with other clay sheets. For example, to simulate the equilibrium separation between clay sheets in a polymer matrix requires that the polymer molecules are free to exchange within the the clay sheet interlayer nanopores and the much larger (reservoir-like) micropores between the clay tactoids (assemblies of clay sheets). The length of simulation required to achieve this equilibration is outside that possible using atomistic molecular dynamics. To overcome this limitation, we use techniques which match the potential of mean force between atomistic and coarse-grained levels, calculated using umbrella sampling techniques. We have optimised coarse-grained potentials to produce identical potential of mean forces to their atomistic equivalent, using umbrella sampling and recombined using the weighted histogram analysis method (WHAM) [8].

To calculate the coarse-grained interaction potentials in the IBI, the inversion of the $g(r)$ to a potential of mean force (PMF) was used. Rather than relying on the $g(r)$ from a single simulation, we can directly calculate the PMF using free-energy calculation methods such as umbrella sampling. Even using biased MD simulations to compute the PMF, the long relaxation times of the polymer molecules required for atomistic simulations of pores- and micropores, means that we performed the computation of the interaction potentials between clay sheet species without any polymer present.

As the only isomorphic substitutions we consider in the clay framework are in the octahedral layer, we assume that the interaction of the clay$_{\text{charge}}$ with other clay coarse-grained particles is identical to that of the neutral pyrophyllite clay$_{\text{basal}}$ particle, since the majority of the interactions will be van der Waals interactions between the clay surface atoms. With this simplifying assumption, we only need to optimise three interaction potentials: clay$_{\text{basal}}$-clay$_{\text{basal}}$, clay$_{\text{edge}}$-clay$_{\text{basal}}$ and clay$_{\text{edge}}$-clay$_{\text{edge}}$.

11.1 Clay$_{\text{basal}}$-clay$_{\text{basal}}$ potentials

An umbrella potential was used to constrain the difference in the z coordinate between two pyrophyllite clay sheets, lying in the xy plane, to a series of values from 8 Å to 20 Å. A very large z lattice parameter was used (120 Å), to ensure the interaction is only between the 2 clay sheets and there is no interaction with images through the z periodic boundary. A picture of this set-up is shown in Figure 19. The black arrow in Figure 19 corresponds to the reaction coordinate of the PMF. The lattice dimensions were 20.685 × 35.9 × 120 Å^3.

Atomistic details:1,000,000 timesteps at 0.5fs for each simulation within each PMF ensemble; 43 such calculations at 0.25 Å intervals (from 8.5 Å to 20 Å). A harmonic constraint of 4.7 kcal mol^{-1} Å^{-2} was used to restrain the clay sheets at their constrained value (the same value for the harmonic restraint was used for all PMF simulations, both atomistic and coarse-grained). The restraint only acts on the difference in z coordinates between the two clay sheets (i.e. the clay sheets are free to move in the xy plane). The same procedure was used for calculating the PMF at the coarse-grained level. **Coarse-grained details**: 256 coarse-grained particles (2 sheets of 128 particles). At each iteration, the update to the potentials used the same procedure as that for the polymer interacting with the clay surface, as described in section 7. The PMF at the atomistic and coarse-grained levels are shown in Figure 19.

11.2 Clay$_{\text{edge}}$-clay$_{\text{basal}}$ potentials

The PMF is calculated for a clay sheet with a 110 edge adsorbing onto a clay sheet lying in the xy plane. A snapshot of this arrangement is shown in Figure 20. The black arrow corresponds to the reaction coordinate.

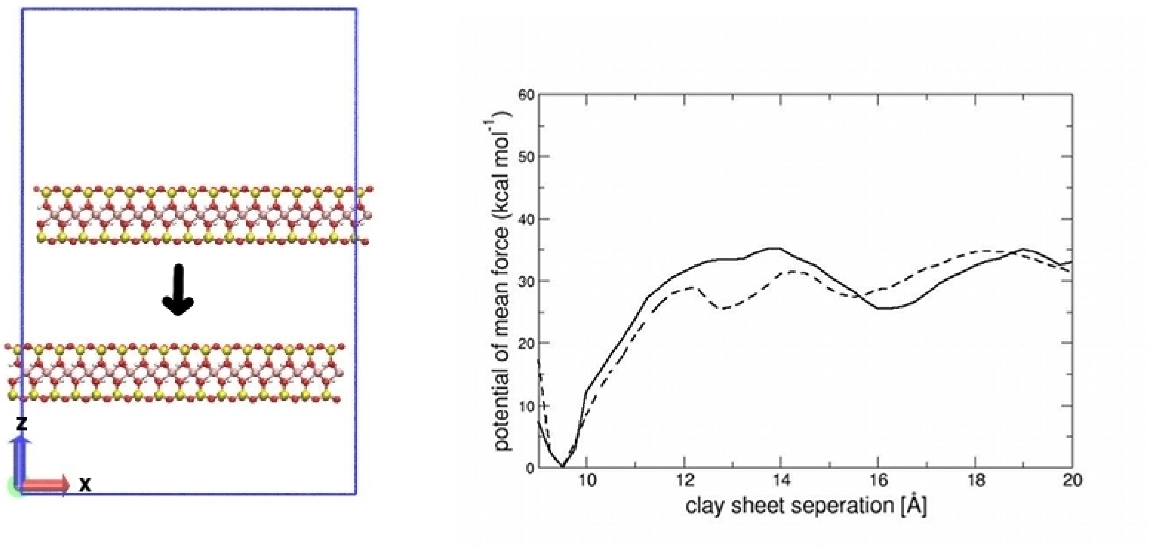

Figure 19: Left panel: an illustration of the potential of mean force calculation between two pyrophyllite clay sheets. The black arrow is the reaction coordinate for the PMF, which corresponds to the difference in z coordinate between the centres of mass of the clay sheets. Right panel: the PMF at the atomistic level (solid line) and the optimised coarse-grained PMF (dashed line).

Atomistic details: the atomistic system contains 1328 atoms with lattice dimensions 20.685 × 35.9 × 85.0 Å^3. The adsorbing clay sheet lies diagonally across the xy plane. The reaction coordinate in figure 20 is the difference in z coordinate between the centre of mass of the adsorbing clay sheet and the clay sheet in the xy plane. 1,000,000 timesteps at 0.5fs for each simulation within each PMF ensemble, 54 such calculations at 0.25 Å intervals (from 14 Å to 27.5 Å). The coarse-grained system contains 128 atoms, including 16 clay$_{\text{edge}}$ atoms. The PMF at the atomistic and coarse-grained levels are shown in Figure 20.

11.3 Clay$_{\text{edge}}$ - clay$_{\text{edge}}$ potentials

The PMF is calculated for 110 edges clay sheets adsorbing onto each other; both clay sheets are lying in the xy plane. A snapshot of this arrangement is shown in Figure 21. The black arrow corresponds to the reaction coordinate. The PMF at the atomistic and coarse-grained levels is shown in Figure 21.

Atomistic details: 1376 atoms. The lattice dimensions are 79.8 × 41.36 × 45.00 Å^3. Both clay sheets lie on the xy plane. 1,000,000 timesteps at 0.5fs for each simulation within each PMF ensemble, 44 such calculations at 0.25 Å intervals (from 25 Å to 36 Å). The PMF at the atomistic and coarse-grained levels are shown in Figure 21.

12 Validation of coarse-grained interaction potentials

One of the major challenges of modelling clay -polymer nanocomposites is reproducing the correct clay swelling behaviour; that is, the changes is spacing between the layers as the amount of intercalated polymer increases. In Figure 22, we show the computed d spacing (*i.e.* the separation between the clay sheets) as the percentage polymer by weight of the system increases, up to 50%. The clay simulated is a montmorillonite and the

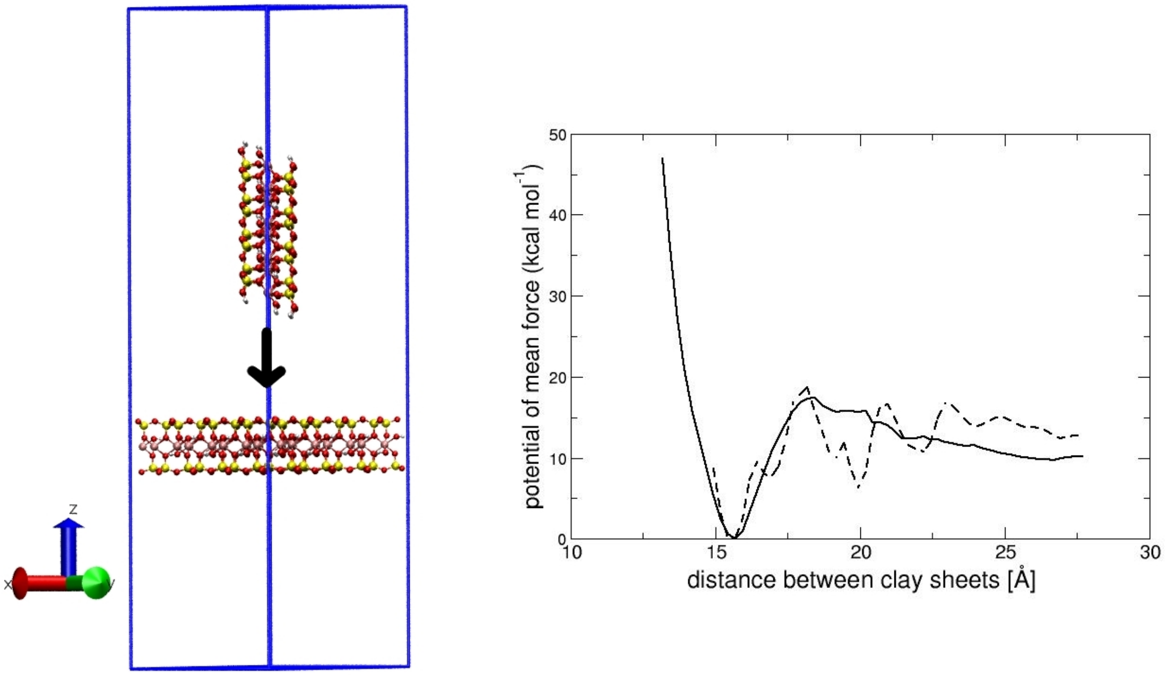

Figure 20: Left panel: an illustration of the potential of mean force calculation between two pyrophyllite clay sheets, with one sheet adsorbing onto the other by its 110 surface. The black arrow is the reaction coordinate for the PMF, which corresponds to the difference in z coordinate between the centres of mass of the clay sheets. Right panel: the PMF at the atomistic level (solid line) and the optimised coarse-grained PMF (dashed line).

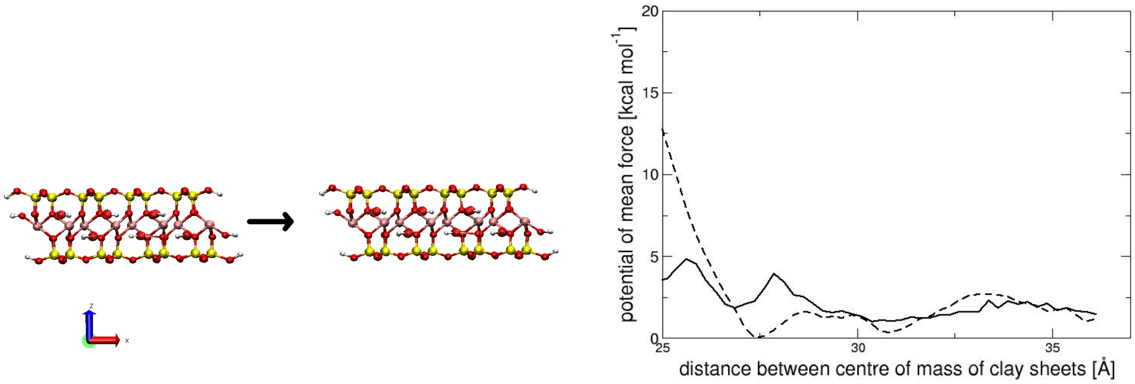

Figure 21: Left panel: an illustration of the potential of mean force calculation (PMF) calculation between two pyrophyllite clay sheets, with each sheet interacting via their (110) surfaces. The black arrow is the reaction coordinate for the PMF, which corresponds to the difference in x coordinate between the centres of mass of the clay sheets. Right panel: the PMF at the atomistic level (solid line) and the optimised coarse-grained PMF (dashed line).

system was simulated at 500K and 1 atmosphere at both atomistic and coarse-grained levels. We find very good agreement at low volume fractions, with identical regions corresponding to the formation of mono-, bi-

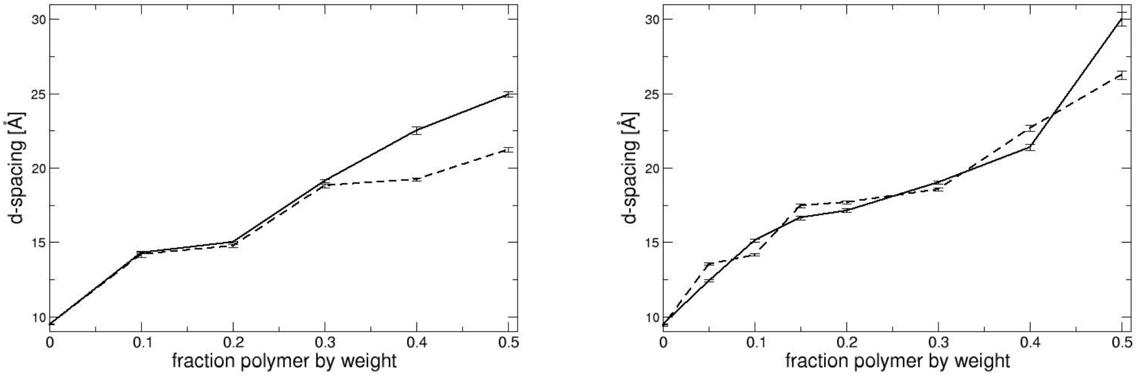

Figure 22: The changes in d-spacing with increased fraction of polymer intercalated between the clay sheets at the atomistic level (black solid lines) and the coarse-grained level (dashed line) for the PVA polymer (left panel) and PEG polymer (right panel). The error bars shown are the standard deviation of the d spacings. We see very good agreement at low polymer factions, correctly representing the transition from mono- to bi- and trilayer of polymer within the clay sheets.

and tri-layer of polymer. Our coarse-grained model has not been fitted to the swelling behaviour, therefore the results of Figure 22 demonstrate that the approach described here of constructing coarse-grained interaction potentials by reproducing the properties of individual interactions in turn, can capture the detailed behaviour of clay-polymer nanocomposites.

13 FabMD Software

We use the FabMD software toolkit, which is a domain-specific approach to manage molecular dynamics simulations across multiple resources. FabMD is based on basic Python libraries (numpy, scipy) and the Fabric library (see http://www.fabfile.org).

The toolkit provides automated administration of input and output directories, and simulation-based workflows in general. It allows users to run sequences of jobs and script execution activities, or combinations using the two, by invoking a one-line command. We have run FabMD-based jobs (which mostly consisted of running LAMMPS) for numerous tasks in this work. For example, we used FabMD to manage the iterative Boltzmann inversion (IBI) required for parameterizing the potentials, allowing us to send IBI runs to available remote resources. The runs that we performed for each IBI iteration typically used 32 cores and lasted between 5 to 25 minutes each.

We aim to package and release FabMD under an open-source license in the near future, and are willing to share the code upon request prior to that time.

References

[1] J. L. Suter, D. Groen, and P. V. Coveney. Chemically specific multiscale modelling of clay-polymer nanocomposites reveals intercalation dynamics, tactoid self-assembly and emergent materials properties. *Adv. Mater.*, submitted, 2014.

[2] R. T. Cygan, J.-J. Liang, and A. G. Kalinichev. Molecular models of hydroxide, oxyhydroxide, and clay phases and the development of a general force field. *J. Phys. Chem. B*, 108(4):1255–1266, 2004.

[3] P. Dauber-Osguthorpe, V. A. Roberts, D. J. Osguthorpe, J. Wolff, M. Genest, and A. T. Hagler. Structure and energetics of ligand binding to proteins: Escherichia coli dihydrofolate reductase-trimethoprim, a drug-receptor system. *Proteins*, 4(1):31–47, 1988.

[4] H. Wang, C. Junghans, and K. Kremer. Comparative atomistic and coarse-grained study of water: What do we lose by coarse-graining? *Eur. Phys. J. E*, 28(2):221–229, 2009.

[5] C.-C. Fu, P. M. Kulkarni, M. S. Shell, and L. G. Leal. A test of systematic coarse-graining of molecular dynamics simulations: Thermodynamic properties. *J. Chem. Phys.*, 137(16):164106, 2012.

[6] K. Johnston and V. Harmandaris. Hierarchical simulations of hybrid polymer–solid materials. *Soft Matter*, 9(29):6696–6710, 2013.

[7] J. L. Suter and P. V. Coveney. Computer simulation study of the materials properties of intercalated and exfoliated poly (ethylene) glycol clay nanocomposites. *Soft Matter*, 5(11):2239–2251, 2009.

[8] M. Souaille and B. Roux. Extension to the weighted histogram analysis method: combining umbrella sampling with free energy calculations. *Comput. Phys. Commun.*, 135(1):40–57, 2001.
